# Epigenomic subtypes of late-onset Alzheimer’s disease reveal distinct microglial signatures

**DOI:** 10.1007/s00401-026-02990-y

**Published:** 2026-02-24

**Authors:** Valentin T. Laroche, Rachel Cavill, Morteza Kouhsar, Joshua Müller, Rick A. Reijnders, Joshua Harvey, Adam R. Smith, Jennifer Imm, Jarno Koetsier, Luke Weymouth, Lachlan MacBean, Giulia Pegoraro, Lars Eijssen, Byron Creese, Gunter Kenis, Betty M. Tijms, Daniel van den Hove, Katie Lunnon, Ehsan Pishva

**Affiliations:** 1 Department of Psychiatry and Neuropsychology, Mental Health and Neuroscience Research Institute (MHeNs), Maastricht University, 6200 MD Maastricht, The Netherlands; 2 Department of Advanced Computing Sciences (DACS), Faculty of Science and Engineering (FSE), Maastricht University, Paul-Henri Spaaklaan 1, 6229 EN Maastricht, The Netherlands; 3 Department of Clinical and Biomedical Sciences, Faculty of Health and Life Sciences, University of Exeter, Royal Devon & Exeter Hospital Barrack Road, Exeter, EX2 5DW UK; 4 Department of Biochemistry, Cardiovascular Research Institute Maastricht (CARIM), Maastricht University, Universiteitssingel 50, 6229 ER Maastricht, The Netherlands; 5 Department of Life Sciences, Brunel University, Kingston Lane, Uxbridge, London, Middlesex UB8 3PH UK; 6 Alzheimer Center Amsterdam, Department of Neurology, Vrije Universiteit Amsterdam, Amsterdam UMC, De Boelelaan 1108, 1081 HV Amsterdam, The Netherlands

**Keywords:** Alzheimer’s disease, Epigenetics, DNA methylation, Subtyping, Microglia

## Abstract

Growing evidence suggests that clinical, pathological, and genetic heterogeneity in late-onset Alzheimer’s disease (LOAD) contributes to variable therapeutic outcomes, potentially explaining many trial failures. Advances in molecular subtyping through proteomic and transcriptomic profiling reveal distinct patient subgroups, highlighting disease complexity beyond amyloid-beta plaques and tau tangles. This underscores the need to expand subtyping across new molecular layers, to identify novel drug targets for different patient subgroups. In this study, we analyzed genome-wide DNA methylation (DNAm) data from three independent postmortem brain cohorts (*N* = 826) to identify epigenetic subtypes of LOAD. We used unsupervised clustering to define subtype-specific DNAm patterns and validated them across cohorts. We then mapped subtype signatures to brain cell types using purified-cell DNAm profiles and integrated bulk and single-nucleus RNA-seq to assess each subtype’s impact on gene expression. Finally, we examined clinical and neuropathological correlates to evaluate biological and clinical significance. We identified two distinct epigenomic subtypes of LOAD, consistently observed across three cohorts. Both subtypes exhibit significant yet distinct microglial methylation enrichment. Bulk transcriptomic analyses highlighted distinct biological mechanisms underlying these subtypes: subtype 1 was enriched for immune-related processes, while subtype 2 was characterized by neuronal and synaptic pathways. Single-nucleus transcriptional profiling of microglia indicated that both subtypes share AD-associated innate-immune remodeling, with subtype differences emerging primarily as state-dependent transcriptional shifts rather than large changes in state abundance. Overall, subtype 1 showed a relative weighting toward more inflammatory microglial programs, whereas subtype 2 showed stronger transcriptional remodeling in specific microglial states alongside relatively greater engagement of regulatory and clearance-associated features. These findings reveal distinct epigenetic and functional microglial states underlying LOAD subtypes, advancing our understanding of disease heterogeneity. This work lays the groundwork for targeted therapeutic strategies tailored to specific molecular and cellular disease profiles.

**Supplementary Information:**

The online version contains supplementary material available at 10.1007/s00401-026-02990-y.

## Introduction

Late-onset Alzheimer’s disease (LOAD) is the most common form of dementia, typically developing after the age of 65 [39]. It primarily affects memory, cognitive function, and behavior. This condition is characterized by the gradual accumulation of amyloid-beta (Aβ) plaques and neurofibrillary tangles (NFTs) in the brain, resulting in the progressive destruction of neurons and brain atrophy [9]. However, significant heterogeneity has been observed among individuals with LOAD in terms of disease onset, progression, symptom variability, spreading of pathology, and their response to treatment [21, 26, 30].

In recent years, molecular subtyping of AD has increasingly leveraged omics data to identify distinct molecular profiles that may contribute to the observed heterogeneity in disease manifestation and mechanisms. This has been particularly transformative as traditional classification methods based on clinical or pathological features alone have proven insufficient. A recent study that used mass spectrometry proteomics to analyze CSF protein profiles identified five distinct molecular subtypes of AD [44]. These subtypes highlight various underlying mechanisms, including neuronal plasticity, innate-immune activation, RNA dysregulation, and dysfunction in the choroid plexus and blood–brain barrier. Another study, which examined the molecular heterogeneity of AD across multiple brain regions, identified five major AD subtypes using transcriptomic data [33]. Importantly, the immune-related and synaptic pathway subtypes predicted in the CSF proteomic are consistent with the transcriptomic-based subtypes of AD. In addition to these shared subtypes, transcriptomic analyses further identified distinct molecular subtypes characterized by alterations in protein metabolism and by the upregulation of genes involved in organic acid metabolic processes, thereby providing additional resolution of LOAD’s molecular heterogeneity.

In addition, the transcriptomic findings revealed subtypes associated with protein metabolism and the upregulation of organic acid-related genes, further enriching our understanding of LOAD’s diverse molecular landscape.

Epigenetic modifications, such as DNA methylation (DNAm) and histone modifications, can alter gene expression without changing the DNA sequence. These modifications are influenced by both genetic predisposition and environmental factors, exerting diverse effects among individuals. Owing to these characteristics, epigenetic modifications serve as valuable molecular markers for identifying the drivers of disease heterogeneity. This is particularly relevant in the context of LOAD, where a complex interplay of genetic, lifestyle, and environmental factors is believed to contribute to the onset, progression, and variability of symptoms [39]. Moreover, an increasing body of evidence suggests that modifiable lifestyle factors, such as physical activity, may delay the onset of dementia [45]. Notably, our previous study highlighted that variation in methylation profile scores associated with lifestyle and environmental factors such as physical inactivity and low educational attainment as predictive markers for the prospective onset of dementia [20]. However, it remains unknown whether distinct DNAm subgroups exist within AD, which could provide further insights into disease heterogeneity.

In the present study, we used genome-wide DNAm data from three independent postmortem brain cohorts to identify epigenomic-based subtypes of LOAD. We employed data-driven methods to identify molecular subtypes of LOAD based on the highest degree of similarity in methylomic patterns within each cohort. The subtypes were further validated through rigorous cross-cohort comparisons using multiple clustering algorithms. To better understand the complex biological mechanisms underlying the observed heterogeneity, we conducted a comprehensive characterization of predicted subtypes at cell-type-specific DNAm levels. In addition, we examined AD risk genes as potential contributors to methylomic heterogeneity and identified transcriptomic correlates for each subtype using both bulk and single-nucleus RNA (snRNA) sequencing data.

## Materials and methods

### Brain samples

We analyzed 835 cortical postmortem brain samples obtained from 3 independent cohorts to investigate epigenomic-based heterogeneity in LOAD. We included 244 prefrontal cortex (PFC) samples from the UK Brain Banks Network (UKBBN), and 220 PFC samples from the University of Pittsburgh’s Alzheimer’s Disease Research Center (PITT-ADRC) [8], and 371 dorsolateral PFC (DLPFC) samples from the Religious Orders Study and Memory and Aging Project (ROSMAP) [2]. Samples were selected from donors with no or minimal AD pathology and definite AD (see Diagnostic Criteria).

### Diagnostic criteria

The diagnosis of LOAD and control samples was established based on clinical and neuropathological data from donors aged over 65 at the time of death. Dementia was defined by an antemortem Mini-Mental State Examination (MMSE) score below 24 or a Clinical Dementia Rating (CDR) score of ≥ 1. Within the dementia group, samples were classified as AD if they met the criteria of a “probable” or “definite” Consortium to Establish a Registry for Alzheimer’s Disease (CERAD) score for neuritic plaques and a Braak neurofibrillary tangle (NFT) stage of ≥ 3. In cases where CERAD data were unavailable, samples with a Braak stage of ≥ 5 were included in the AD group.

Control samples were defined using stringent criteria specific to each brain bank. They were required to have a Braak stage of 0–2 for NFT pathology and negative for amyloid (CERAD neuritic plaque score 0). In addition, when available from brain banks, both AD and control samples were screened for co-occurring other tau pathologies, including argyrophilic grain disease, as well as alpha-synuclein and TDP-43 pathologies. Only samples negative for these co-pathologies, with none to moderate cerebrovascular disease and rare microinfarcts, were included. Furthermore, samples from donors with known systemic hematopoietic malignancies, such as leukemia, were excluded to prevent the inclusion of malignant cells circulating in the brain.

### Methylomic profiling and data harmonization

For the UKBBN and PITT-ADRC cohorts, genomic DNA was extracted from 30 mg of fresh-frozen tissue using the AllPrep DNA/RNA/miRNA Universal Kit (QIAGEN), followed by bisulfite treatment with the EZ-96 DNA Methylation-Gold Kit (Zymo Research). The treated DNA was analyzed using Illumina’s Infinium MethylationEPIC v1.0 BeadChip arrays, quantifying DNAm at over 850,000 CpG sites. For the ROSMAP cohort, Illumina 450K methylation raw data files were obtained from the Accelerated Medicine Partnership (AMP-AD) portal (synID: syn7357283). Uniform and stringent quality control (QC) and normalization procedures were then applied across all three cohorts.

The raw methylation IDAT files were imported into R (version 4.3.0) using the ‘readEPIC’ function from the wateRmelon R package (v.2.6.0) [35], generating “MethylumiSet” objects. For quality control (QC), samples with a median signal intensity below 1000 were excluded. Additional outliers were removed using the ‘outlyx’ function, which detects anomalies based on the inter-quartile range (IQR) and Mahalanobis distance. Bisulfite conversion efficiency was assessed with the ‘bscon’ function, discarding samples with efficiency below 0.8. The pfilter function was used to remove samples in which more than 1% of probes had a detection *p* value > 0.05. As a result, eight UKBBN samples were removed. To further control data quality, probes with a detection *p* value > 0.05 in at least 5% of samples and/or a bead count < 3 in at least 5% of samples were excluded across all samples.

Methylation-inferred sex was estimated using the estimateSex function based on CpG sites on chromosomes X and Y. One UKBBN sample was excluded due to a discrepancy between methylation-inferred and recorded sex. Data normalization was performed using ‘BMIQ’ (Beta-Mixture Quantile intra-sample normalization), preserving inter-sample variance. Probe-Wise Outlier Detection (PWOD) via the ‘homonymous’ function removed probes associated with known variants to prevent downstream biases. Additional filtering excluded probes with SNP IDs, cross-hybridizing probes [28], and those on sex chromosomes.

Cell-type composition in bulk PFC DNAm data was computed using CETYGO (v0.99.0) [47], employing a reference panel to distinguish inhibitory (GABAergic) neurons, excitatory (glutamatergic) neurons, oligodendrocytes, microglia, and astrocytes. To account for mutual technical and biological effects (e.g., age, sex, postmortem interval, plates, cell composition, and cohort-specific batches), a linear multiple regression model was applied to each methylation probe. Non-variant probes were filtered using the 90th percentile absolute deviation, resulting in 325,834 CpGs from EPIC arrays for subtyping. In the ROSMAP cohort, the CpG set was defined by the overlap between the processed 450K array and the available 450K probes within the final EPIC CpGs, yielding 148,951 CpGs.

Following pre-processing and QC, the final dataset included 235 UKBBN samples (124 LOAD, 111 controls), 220 PITT-ADRC samples (192 LOAD, 28 controls), and 371 ROSMAP samples (248 LOAD, 123 controls). This dataset, detailed in Supplementary Table 1, integrates diverse demographics, clinical profiles, and neuropathological information.

### Bulk RNA sequencing

Total RNA quality in the UKBBN and PITT-ADRC cohorts was assessed using the Agilent 4200 TapeStation System, with samples having an RNA Integrity Number (RIN) below 3 excluded. Library preparation was conducted using the Illumina Stranded mRNA Prep kit, followed by sequencing on the NovaSeq 6000 platform at the Exeter Sequencing Service. Raw sequencing data in FASTQ format underwent quality trimming to remove low-quality bases and adapter sequences. Processed reads were aligned to the human reference genome (GRCh38) using the STAR aligner (v2.7.3a) [10] with default parameters. After alignment, a raw count matrix was generated, and genes with low expression, defined as counts below 10 in over 80% of samples. Gene counts were normalized using the Trimmed Mean of M-values (TMM) method from the edgeR package (v3.42.4) [37]. The normalized counts were then transformed into log-transformed counts per million (logCPM) to stabilize variance and improve interpretability.

### Genotyping, imputation, and generation of polygenic scores

Genotyping was conducted using the Illumina Global Screening Array (GSA) for the UKBBN and PITT-ADRC cohorts. For the ROSMAP cohort, genotyping data from the Affymetrix GeneChip 6.0 (Affymetrix, Inc.) and the Illumina HumanOmniExpress chip array were obtained from (https://www.synapse.org/). Quality control was performed using PLINK (v2.0), excluding samples with > 5% missing values and SNPs with > 1% missing values. In addition, SNPs with a minor allele frequency (MAF) < 0.05 and a Hardy–Weinberg equilibrium *p* value < 1.0 × 10^−3^ were filtered out to retain the most reliable signals.

Processed genotype data were merged with the 1000 Genomes Project dataset, and principal component analysis (PCA) was performed to determine sample ethnicity. Samples of non-European ancestry were removed based on PCA results. The quality-controlled genotype data were uploaded to the Michigan Imputation Server and imputed using Minimac4 with the 1000 Genomes reference panel (phase 3, version 5). The imputed data for each chromosome were compiled into a unified VCF file using BCFtools (v1.9) [7] with tri-allelic SNPs removed using VCFtools (v0.1.16) [6]. The final VCF file was converted into PLINK binary format.

**Fig. 1:**
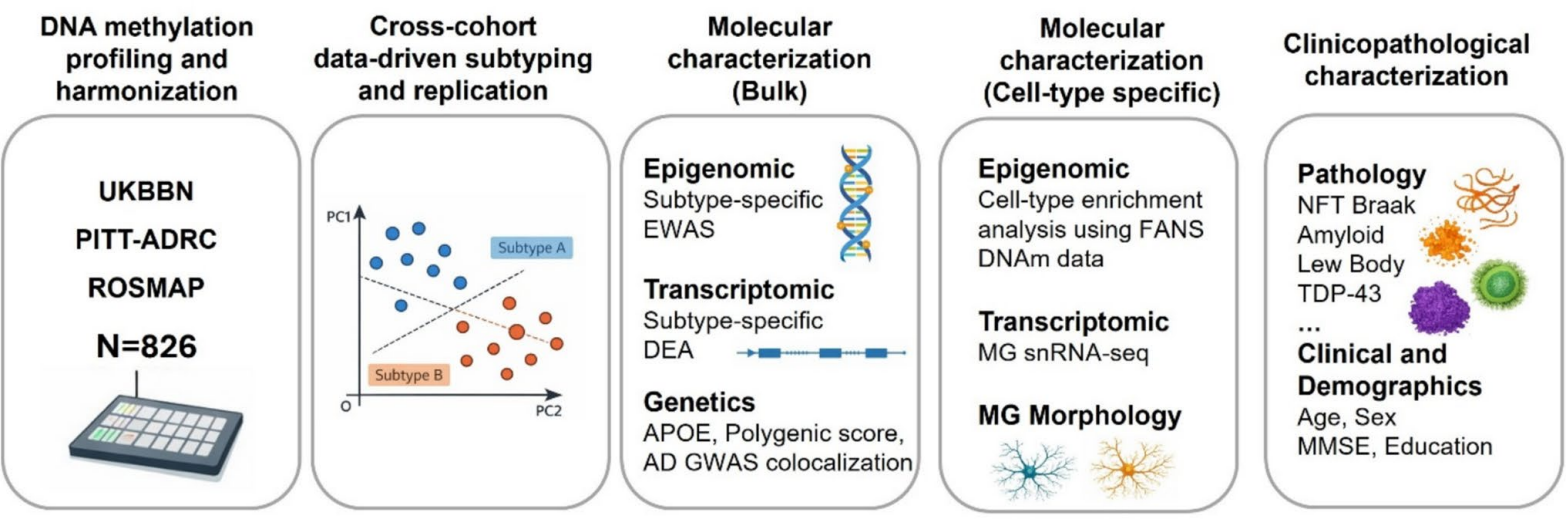
Study design and analytical framework. Cortical DNA methylation (DNAm) data from three independent cohorts (UKBBN, PITT-ADRC, and ROSMAP; *N* = 826) were harmonized and used for cross-cohort, data-driven subtyping of LOAD. Identified subtypes were replicated across cohorts and subsequently characterized using bulk epigenomic, transcriptomic, and genetic analyses, followed by cell-type-specific molecular profiling incorporating FANS-derived DNA methylation data, single-nucleus RNA (snRNA) sequencing, and quantitative microglial (MG) morphology. Finally, DNAm-defined subtypes were linked to neuropathological features and clinical and demographic measures

Polygenic scores (PGS) were generated using the imputed genotype data and PRSice-2 software [5]. Genetic variants associated with AD were selected based on the genome-wide association study (GWAS) by Bellenguez et al. [1] (Fig. 1).

### Data analysis

#### Clustering algorithms

To identify cortical brain subgroups with similar DNAm profiles, agglomerative hierarchical clustering using the Ward D2 method with Euclidean distance and K-means clustering as a nonhierarchical approach were applied to LOAD samples, with each cohort analyzed independently. The optimal number of clusters for both methods was determined using the Elbow method. To assess clustering consistency, Normalized Mutual Information (NMI) analysis was performed for each cohort using the Aricode R package (v1.0.3) [50] (https://github.com/jchiquet/aricode), comparing hierarchical and K-means clustering results. The cluster number with the highest NMI value was selected for further analysis.

To assess cluster specificity, Sparse Partial Least Squares Discriminant Analysis (sPLSDA) was performed using the mixOmics package (v6.24.0) [38] for each clustering algorithm to identify the most discriminative features. From each sPLS-DA model, 12,000 features corresponding to 6 components were extracted. To evaluate robustness, cluster labels were randomized 10 times across population proportions ranging from 100 to 1%, followed by repeated sPLS-DA modeling for each iteration. The overlap between the 12,000 extracted features from each iteration and those from the original cluster labels was then examined. Technical batch effects, including plate and chip variations, were assessed using Spearman’s correlation test for continuous variables and Normalized Mutual Information (NMI) for discrete variables, the latter being particularly suited for evaluating clustering outcomes.

To verify the generalizability of findings across Illumina EPIC arrays (UKBBN and PITT-ADRC cohorts) and 450K arrays (ROSMAP cohort), Weighted Gene Co-expression Network Analysis (WGCNA) was employed. Co-expression networks were constructed using the ‘blockwiseModules’ function in the WGCNA R package (v1.72-5) [22] based on selected EPIC array probes. Module eigenprobes were then calculated using the ‘moduleEigengenes’ function, and their associations with categorical cluster labels were assessed through ANOVA followed by post hoc Tukey’s tests. For modules showing significant differences between identified clusters, eigenprobes were recalculated using the subset of probes available on the 450K array. Cluster-associated co-methylated probe preservation was confirmed by demonstrating that eigenvalue differences between cluster labels remained significant with the 450K probes and that eigenvalue differences for specific subtypes across both platforms were non-significant.

#### Cross-cohort replication

To identify matching clusters with maximum similarity in DNAm profiles across the UKBBN, PITT-ADRC, and ROSMAP cohorts, we employed two complementary approaches at both the probe and sample levels.

In the first approach, we calculated the median DNAm value per probe for each clustered brain sample within each cohort. Pearson correlation tests were then used to assess the relationship between these median values across the three cohorts.

In the second approach, the UKBBN cohort was designated as the discovery dataset. Using the ‘sPLSDA’ function from the mixOmics package (v6.24.0) [38], we reduced data into a lower dimensional latent space while preserving class separation. The first two latent spaces of sPLSDA were mapped against the clusters identified in this cohort. Convex hulls were established around each UKBBN cluster by connecting the outermost points. DNAm profiles from a replication cohort (either PITT-ADRC or ROSMAP) were then projected onto these latent spaces, and the number of replication cohort samples falling within the UKBBN cluster hulls was recorded. To assess significance, contingency tables were constructed, and a hypergeometric test was performed to determine overlaps between the discovery and replication cohort clusters. This procedure was repeated with PITT-ADRC and ROSMAP serving as discovery sets, while the other two cohorts acted as replication cohorts. An epigenomic-based LOAD subtype was confirmed only when both approaches validated the DNAm profile similarities across all three cohorts, at both the probe and sample levels, using two clustering methods.

#### Subtype-specific epigenome-wide association analysis

Epigenome-wide association studies (EWASs) were conducted using multiple linear regression models to identify differentially methylated positions (DMPs) associated with each predicted subtype in each cohort. Methylation data were rigorously adjusted for potential confounders, including age, sex, cell-type composition, brain banks, and plates, ensuring consistency with previous clustering steps. Surrogate variable analysis (SVA) was applied to adjust for unwanted variations unrelated to the main outcome, further refining accuracy. *p* value inflation was assessed using the inflation index for each EWAS, and estimates and standard errors were corrected if needed using the Bacon package in R (v1.28.0) [46] before proceeding to the meta-analysis. Bacon applies an empirical Bayes approach to model and remove bias and inflation in test statistics, thereby improving the accuracy of effect size estimates in high-dimensional datasets. The meta-analysis was performed using the inverse variance method via the rma.uni function in the metafor R package (v4.6-0) [48]. Subtype-specific DNAm signatures were identified using stringent criteria. Specifically, DMPs were required to have a Bonferroni-adjusted *p* value under 0.05. The overlap between DMPs identified across subtypes in the meta-analysis was assessed using the geneOverlap R package (v1.40.0) (http://shenlab-sinai.github.io/shenlab-sinai/). To identify CpGs located in active regulatory chromatin, we additionally overlapped CpG positions with cell-type-specific H3K4me3 and H3K27ac ChIP-seq peaks from Nott et al. [34], applying FC > 2 and FDR < 0.05 to define significant regions. For each LOAD subtype (LOAD, LOAD-S1, LOAD-S2, Unassigned), we quantified the proportion of CpGs overlapping each regulatory feature and tested enrichment relative to the background (148,951 CpGs) using Fisher’s exact tests with Benjamini–Hochberg (BH) correction.

#### Cell-type-specific DNAm enrichment analysis

To investigate the cell-type specificity of the methylation signatures associated with the identified subtypes, we employed the CEAM (Cell-type Enrichment Analysis for Methylation) framework developed by Müller et al. [32] (https://um-dementia-systems-biology.shinyapps.io/CEAM/). CEAM uses fluorescence-activated nuclei sorting (FANS)-derived methylation profiles from purified brain cell populations, neurons, oligodendrocytes, microglia, and astrocytes, to construct cell-type-specific CpG sets. These CpG sets are stratified into three specificity levels, ranging from uniquely cell-type-specific CpGs to those shared across multiple cell types, enabling nuanced enrichment analyses. For each LOAD subtype, meta-analysis-derived DMPs were independently assessed against these CpG panels.

#### Co-localization analysis

To investigate whether subtype-specific methylation signals share a common genetic basis with AD genetic risk loci, we performed co-localization analysis using methylation quantitative trait loci (mQTL) data from the ROSMAP cohort, accessed via Brain xQTLServe (https://mostafavilab.stat.ubc.ca/xqtl/). As input, we extracted cis-mQTLs (*p* < 1.0 × 10^−5^) associated with DMPs identified in the subtype-specific EWASs, using the suggestive nominal *p* value threshold of 1.0 × 10^−5^. Bayesian co-localization was conducted using the coloc R package (v5.2.3) [51] for all pairwise comparisons between genomic regions associated with AD risk (as identified by Bellenguez et al.) [1] and variants linked to LOAD subtype DMPs. The coloc.abf function was used, and co-localization was considered significant when the combined posterior probability of hypotheses H3 and H4 exceeded 0.90.

#### Bulk transcriptomic analysis

Differential expression analysis (DEA) was performed using the Limma R package (v3.55.10) [36], comparing LOAD subtypes to their respective controls across the three cohorts. To account for potential confounders, the analysis included covariates such as age, sex, RIN, brain banks, and surrogate variables, which were incorporated to adjust for unmeasured variation, including differences in cell-type composition. Bacon correction was applied to estimates and standard errors to reduce bias. Genes were considered differentially expressed if they showed a consistent direction of effect across the ROSMAP, UKBBN, and PITT-ADRC cohorts, a fold change > 1.5 in at least one cohort, nominal significance in all three cohorts (*p* < 0.05), and FDR-adjusted significance in at least one cohort (adjusted *p* < 0.05). GO enrichment analysis was conducted using the clusterProfiler R package (v4.12.6) [31] to identify biological processes, molecular functions, and cellular components significantly associated with DEGs for each subtype.

In addition, we integrated DNA methylation and mRNA expression data from all three cohorts to examine CpG–gene regulatory relationships in two LOAD subtypes. Subtype-specific CpGs were obtained from prior analyses and matched to processed β-values and log2-CPM expression data. Regulatory regions were defined as transcription start site (TSS) ± 10 kb using Ensembl GRCh38 gene models (retrieved via *biomaRt*). CpG coordinates provided by Illumina manifest were lifted from hg19 to hg38, and genomic overlaps between CpGs and extended promoter regions were identified using *GenomicRanges*. Spearman correlations between CpG and mRNA expression were computed separately for LOAD-S1 and S2. *p* values were adjusted using the BH method, and associations with FDR < 0.1 at least in two cohorts were considered significant.

#### Single-nucleus microglial transcriptomes analysis

Subtype-specific analyses of gene expressions across 12 microglial states were conducted using snRNA-seq data from the ROSMAP study. This dataset comprises 425 samples, including 70 definite AD cases and 64 control samples, overlapping with the subset of ROSMAP data utilized in this project. We examined the single-nucleus transcriptomic signatures of our identified subtypes using the 12 microglial states characterized by Sun et al. [43].

*Proportional abundance analysis:* For each donor’s sample, we extracted the number of single nuclei assigned to each microglial state and calculated the proportional abundance of each state relative to the total number of microglial cells in that sample. Analyses focused on proportional microglial-state abundance, as raw cell counts are confounded by sampling depth and technical variability. To ensure stability of proportional estimates, we excluded any microglial state in which fewer than 100 cells were present in any group. Group differences in proportional abundance were assessed using a one-way ANOVA, followed by Tukey’s HSD post hoc tests.

*Assessment of global subtype-specific microglial expression shifts using PCA:* To evaluate subtype-specific shifts in global microglial gene-expression patterns, we used pre-generated pseudobulk snRNA-seq expression matrices for each microglial state across controls, LOAD-S1, and LOAD-S2. Expression data were normalized using the TMM method and log-transformed prior to analysis. Samples were separated by groups, and only the control samples were used to define the PCA structure. Before PCA, control samples were screened for outliers. Outlier detection was performed by computing principal components on the control expression matrix and calculating each sample’s distance from the median. Samples exceeding a median + 3.5 × MAD threshold were removed, and PCA was re-run on the cleaned control subset. The cleaned control matrix was then gene-wise centered and scaled. PCA was performed on this standardized control dataset, yielding a rotation matrix (loadings) and control component scores. The first two principal components and their explained variance were retained for visualization. Next, to enable direct comparison across groups, LOAD-S1 and LOAD-S2 samples were transformed using the same gene-wise mean and standard deviation estimated from the control samples. These scaled matrices were projected into the PCA space by multiplying them with the control PCA rotation matrix. This produced component scores for control, LOAD-S1, and LOAD-S2 in a shared coordinate system, allowing the distributions of each group to be visualized and compared. Statistical significance of group separation in PCA space was assessed using permutation testing (10,000 permutations). Median inter-group distances were compared against empirical null distributions, with 95% confidence intervals derived from permutation distributions and *p* values corrected for multiple testing using the BH procedure.

*Microglial activation score and Z scores:* For each microglial state, we quantified microglial program activity using a denoised eigengene score derived from the state’s marker genes. After TMM normalization and log-transformation, we extracted the logCPM expression matrix for the marker genes associated with each MG state (downloaded from https://compbio.mit.edu/microglia_states/). For each state, we performed PCA across samples on the marker-gene-expression matrix and used the first principal component (PC1) as a per-sample activation score, capturing the dominant axis of coordinated variation across the marker set. To ensure interpretability, the sign of PC1 was oriented such that higher scores corresponded to higher average expression of the marker genes. To compare activation across groups, scores were standardized into *Z* scores relative to controls. Group differences in microglial activation between S1 and S2 were assessed by comparing the corresponding *Z* scores using a two-sample *t* test (two-sided). This test evaluated whether the mean standardized activation scores differed significantly between the two groups for each microglial state.

*Differential expression analysis:* The muscat R package (v1.14.0) was used with default parameters. Non-expressed or lowly expressed genes were filtered out, and outlier detection was performed at the cell level. To generate a total expression value for each sample, cells were aggregated in a pseudobulk manner by summing RNA expression from all cells within each microglial state. Pseudobulk DEA was then conducted for each microglial state, comparing each subtype both to healthy controls and to one another. Genes were considered significant if they were differentially expressed in subtype-to-subtype comparisons and also differentially expressed relative to controls in at least one subtype. A more lenient significance threshold (*p* < 0.05) was used as a complementary approach to dimensionality-reduction-based snRNA-seq analyses, to aid interpretation of subtype-specific transcriptional patterns rather than for definitive gene-level inference.

#### Microglial morphology analysis

To explore whether the DNA methylation-defined LOAD subtypes differ in microglial phenotypes in situ, we leveraged quantitative microglial morphology data available for a subset of ROSMAP participants through the AMP-AD Knowledge Portal (syn25006903). Specifically, we analyzed microglial Stage 3 pathology measures, corresponding to activated microglia, and derived the proportion of activated microglia (PAM) following the established framework described by Felsky et al. [11].

PAM and Stage 3 microglial measures were examined across four brain regions with available data: inferior temporal cortex, midfrontal cortex, ventral medial caudate, and posterior putamen. In addition to LOAD-S1 and LOAD-S2, and Unassigned cases, this analysis included an asymptomatic Alzheimer’s disease (AsymAD) group, defined as individuals who were cognitively unimpaired at the time of death but exhibited neuropathological evidence of AD. Pairwise comparisons between groups were performed using two-sample Welch’s *t* tests to account for unequal variances. Effect sizes were quantified using Cohen’s *d* to assess the magnitude and direction of group differences independent of sample size. All analyses were conducted on a region-wise basis.

## Results

### Data-driven clustering

The clustering analysis was conducted on 325,834 CpGs from EPIC arrays and 148,951 CpGs from 450K arrays, following stringent pre-processing and filtering steps. Clustering algorithms were applied exclusively to samples with a definite LOAD diagnosis, initially identifying three to five optimal clusters across the three cohorts when evaluated using the Elbow method (Supplementary Fig. 1). Further validation with the NMI test demonstrated high similarity between hierarchical and K-means clustering methods, with the strongest agreement observed when selecting three clusters (Supplementary Table 2). To maintain consistency across cohorts, clusters within each dataset were randomly labeled as A, B, and C.

The specificity of the detected clusters was further validated by randomizing cluster labels across varying proportions of the sample populations. Minimal overlap was observed between the features distinguishing the original clusters and those identified under randomized labels, particularly when randomization was applied to the entire dataset. This finding indicates that the original clusters are distinct and unlikely to result from random assignment, reinforcing the robustness and biological relevance of the detected clusters (Supplementary Fig. 2).

No significant association was observed between the identified clusters and known technical batch effects, including plate variations and brain sample sources (Supplementary Table 3). This confirms the reliability of the clustering results, suggesting that the detected clusters are more likely to reflect true biological differences rather than procedural inconsistencies. The distribution of samples assigned to each cluster using both clustering methods across the three cohorts is reported in Supplementary Table 4.

Illumina EPIC arrays were used for DNAm quantification in the UKBBN and PITT-ADRC cohorts, covering over 850K CpG sites, while the ROSMAP cohort utilized the 450K array. To assess the generalizability of findings across these two array types, WGCNA was applied. The significant relationship between module eigenvalues and the three identified clusters, as determined by ANOVA, remained preserved when reconstructing modules using only the subset of CpGs available on 450K arrays. In addition, no significant changes in module eigenvalues within the same cluster were observed when transitioning from EPIC to 450K arrays, supporting the robustness and cross-platform reproducibility of the clustering results (Supplementary Figs. 3–6).

### Cross-cohort replication of epigenomic-based subtypes

Cross-cohort replication analyses identified two distinct subtypes of LOAD based on DNAm profiles measured in bulk cortical brain samples. Replication was conducted using two complementary strategies at the CpG and sample levels.

**Fig. 2:**
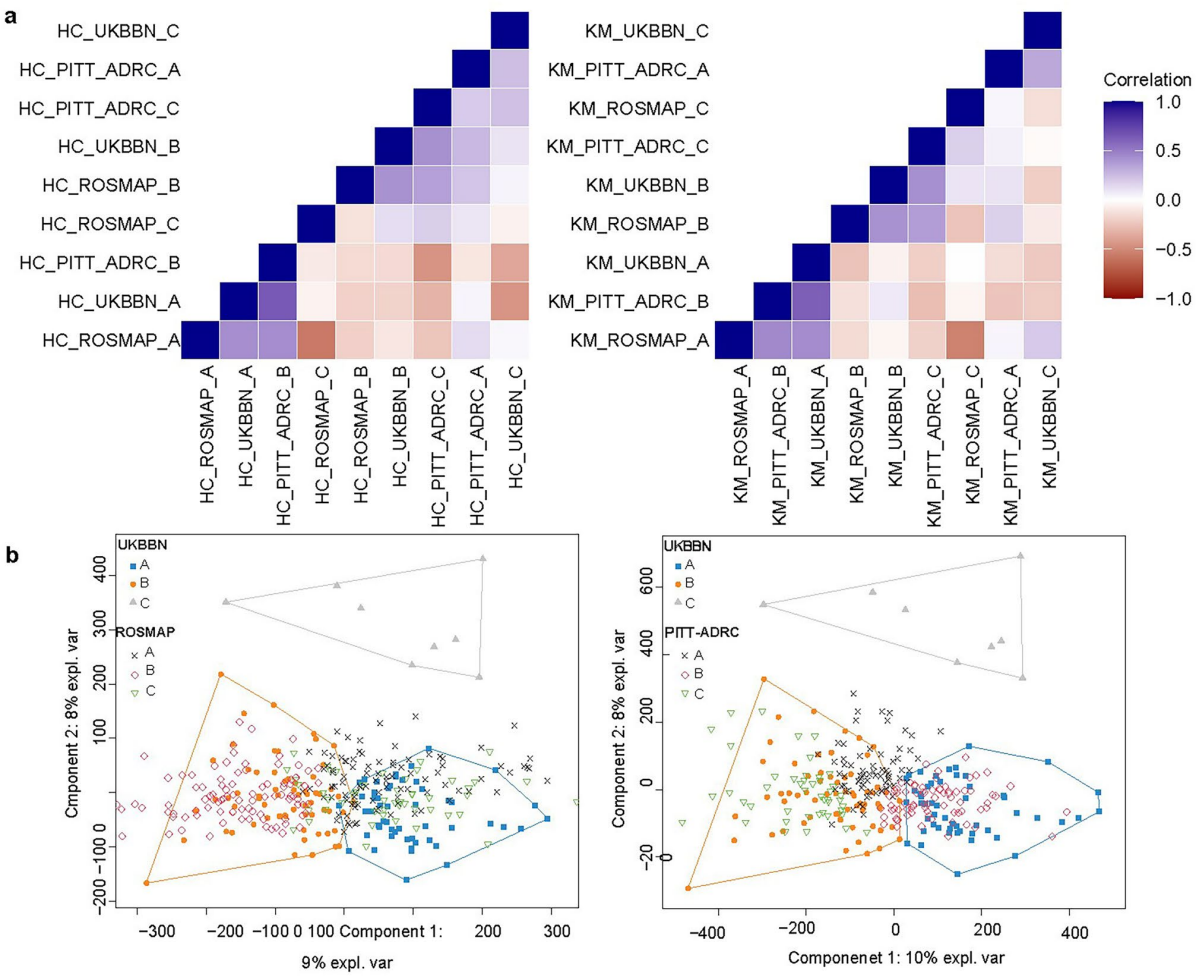
Cross-cohort generalizability of DNAm-based clusters. **a** Visual representation of the correlation-based replication of the identified clusters across three cohorts using hierarchical (left), and K-means (right) methods. The first block (LOAD-S1) includes UKBBN cluster A, PITT-ADRC cluster B, and ROSMAP cluster A, while the second block (LOAD-S2) includes UKBBN cluster B, PITT-ADRC cluster C, and ROSMAP cluster B. **b** Spatial overlap analysis showing cluster assignments across the three cohorts. In this example, the clusters were confirmed through iterative projections, where UKBBN cohort’s first two latent spaces were projected onto the PTT-ADRC and ROSMAP to verify spatial overlap across datasets

CpG-level replication assessed the correlation between median DNAm values per CpG for each data-driven cluster across the three cohorts. This analysis revealed two distinct blocks of correlated clusters: the first block included UKBBN cluster A, PITT-ADRC cluster B, and ROSMAP cluster A, while the second block comprised UKBBN cluster B, PITT-ADRC cluster C, and ROSMAP cluster B. Detailed heatmap plots illustrating the associations between clusters using both methods are provided in Fig. 2a.

The second replication analysis examined the spatial overlap of the distinct clusters across the three cohorts using latent spaces. Matching clusters across independent cohorts were confirmed by iteratively swapping the study samples, where the latent spaces from one cohort were used to project the other two. Notably, the two blocks of clusters identified through correlation analysis were further validated, showing significant overlap in latent spaces, as assessed by Fisher’s tests. An example projection of UKBBN latent spaces onto PITT-ADRC and ROSMAP datasets is illustrated in Fig. 2b, with detailed Fisher’s test statistics provided in Supplementary Table 5. In both analyses, no matching clusters were identified for UKBBN cluster C, PITT-ADRC cluster A, and ROSMAP cluster C.

**Fig. 3:**
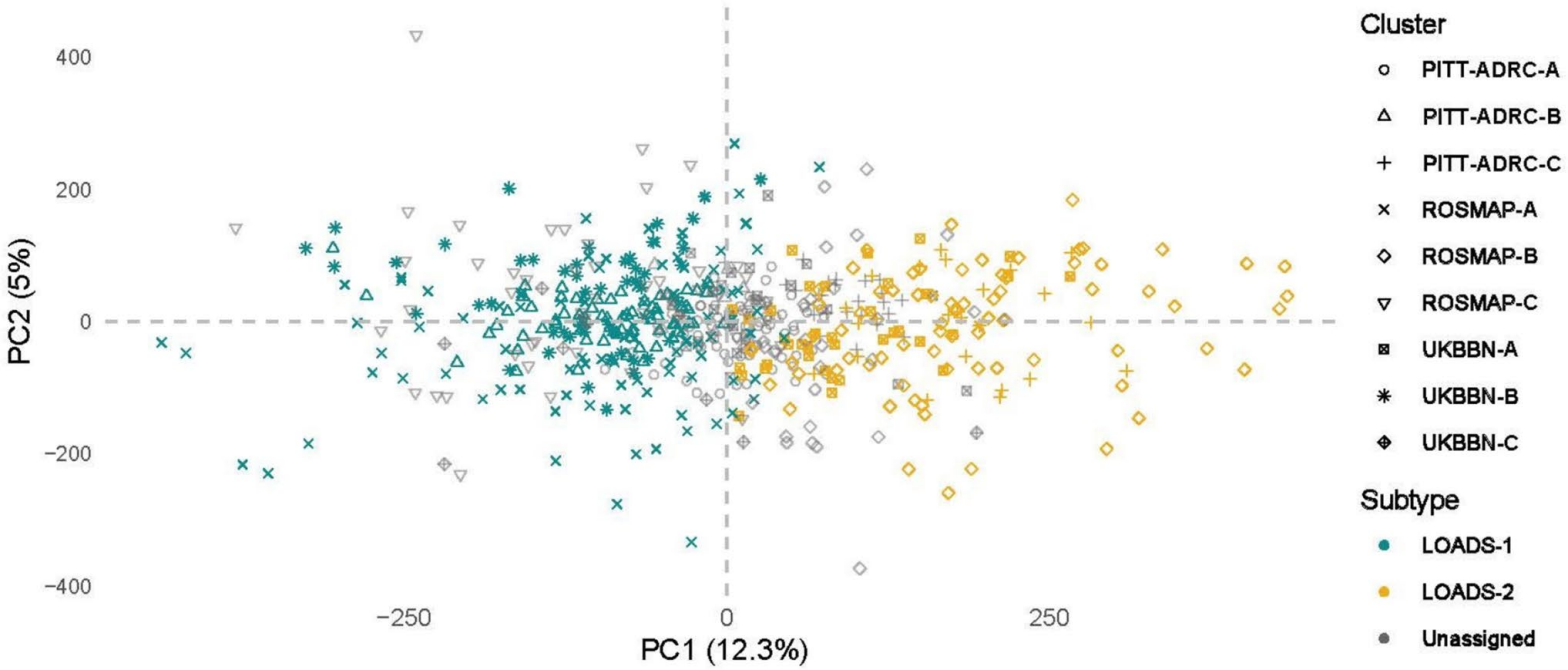
Characterization of the methylomic signatures of predicted LOAD subtypes. Visualization of the first two PCs for LOAD subtypes across all cohorts. Plot of the first two PCs (PC1 and PC2) for DNAm profiles from all samples in the UKBBN, PITT-ADRC, and ROSMAP cohorts. Samples are labeled according to their subtype assignments: Subtype 1 (LOAD-S1) and Subtype 2 (LOAD-S2). ‘Unassigned’ samples are also indicated, representing clusters without matching correlations across cohorts

As a result, these clusters were labeled as ‘Unassigned’ samples for further analysis. This classification remained consistent across both hierarchical and K-means clustering methods, reinforcing the robustness of the clustering results across different methodologies. To ensure subtype consistency, only samples consistently labeled as a subtype across both clustering methods were retained, with the final counts for each subtype reported in Supplementary Table 6. From this point forward, the first block of clusters is referred to as Subtype 1 (LOAD-S1) and the second block as Subtype 2 (LOAD-S2). Figure 3 presents a plot of the first two principal components (PCs) for the pooled dataset comprising UKBBN, PITTADRC, and ROSMAP cohorts, with samples labeled according to their confirmed LOAD subtypes.

### Subtype-specific EWAS

The distinct methylomic signatures of the newly defined LOAD subtypes (LOAD-S1 and LOAD-S2), the Unassigned group, and overall LOAD were characterized using EWAS across the UKBBN, PITT-ADRC, and ROSMAP cohorts, followed by meta-analyses. These analyses focused on 148,951 highly variable CpGs shared between the EPIC and 450K arrays, which were also used in the clustering algorithms. Comparing DNAm profiles of healthy controls (HC) with overall LOAD identified 461 DMPs. In addition, 70 DMPs were associated with LOAD-S1, 323 with LOAD-S2, and 182 with the Unassigned group. DMPs were considered significant if they met the criteria of Bonferroni-adjusted *p* value 0.05 (Supplementary Tables 7–10).

LOAD-S1 and LOAD-S2 showed distinct DNAm profiles, with the least overlap (one shared CpG), and this overlap was not statistically significant. The greatest overlap with overall LOAD DMPs was observed in the Unassigned group (OR 450.60), followed by LOAD-S2 (OR 210.26) and LOAD-S1 (OR 36.30). These findings highlight that LOAD-S1 and LOAD-S2 represent the most distinct DNAm profiles. Similarities and differences between LOAD subtypes and the Unassigned group relative to overall LOAD DMPs are detailed in Supplementary Table 11, with CpG overlaps visualized in Supplementary Fig. 7.

Subtype-specific CpGs showed clear differences in their regulatory distribution across cell types. LOAD-S2 CpGs were significantly enriched in microglial promoters and H3K27ac-marked microglial regulatory regions (FDR < 0.05), whereas LOAD-S1 CpGs showed no significant enrichment in any promoter, enhancer, or H3K27ac feature after multiple testing correction. LOAD (subtype-combined) CpGs demonstrated modest enrichment in astrocytic and neuronal enhancers, while unassigned CpGs did not show enrichment in any regulatory category. These findings indicate that LOAD-S2 methylation changes preferentially localize to microglia-specific active regulatory elements. Full regulatory annotations and enrichment statistics are provided in Supplementary Table 12, and odds ratio estimates with significance labels are shown in Supplementary Fig. 8.

To evaluate the relationship between subtype-specific methylomic signatures identified in this study and previously reported DMPs associated with AD pathology, we examined the overlap of significant DMPs linked to neurofibrillary tangles (NFTs) in the PFC as reported by our group [42]. Of the 236 Bonferroni-significant DMPs linked to AD pathology in that study, 150 CpGs were included in the subset analyzed in the current study. The greatest overlap was observed with overall LOAD (73 CpGs; OR 360.24) and the Unassigned group (48 CpGs; OR 516.16). Notably, LOAD-S2 displayed a greater overlap with previously reported DMPs from the PFC DNAm meta-analysis (34 CpGs; OR 150.01), whereas the overlap with LOAD-S1 was minimal (one CpG) and did not reach statistical significance (Supplementary Table 13).

### Cell-type-specific methylation and LOAD subtypes

To investigate the relationship between LOAD subtypes and brain cell types, we used the CEAM framework to assess enrichment of DMPs in cell-type-specific CpG sets derived from purified brain cells. Overlap analyses revealed significant enrichment of DMPs from both LOAD-S1 and LOAD-S2 subtypes in microglia, with odds ratios of 4.77 and 4.95 (highest specificity level), respectively. Notably, there was minimal overlap between the microglial methylation signatures of LOAD-S1 and LOAD-S2, with only one DMP shared, suggesting distinct microglial epigenetic profiles despite their shared association with this cell type (Supplementary Table 14).

### AD genetics and epigenomic-based subtypes

To investigate the contribution of Alzheimer’s disease (AD) genetic risk to epigenomic heterogeneity in LOAD, we examined AD risk loci in relation to subtype-specific differentially methylated positions (DMPs). Subtype-specific DMPs were mapped to cis-mQTLs derived from the ROSMAP Brain xQTLServe database and tested for shared causal variants with AD risk regions using Bayesian co-localization analysis. Evidence for co-localization (PP [H3 + H4] > 0.90) was observed for distinct genomic loci across subtypes. Specifically, DMPs associated with overall LOAD co-localized with AD risk variants at the BIN1 locus, whereas LOAD-S2-specific methylation signals showed co-localization with variants at the SPI1 locus. In addition, co-localization at the MAPT locus was observed for DMPs identified in association with the Unassigned group. Detailed co-localization statistics are provided in Supplementary Table 15.

### Distinct transcriptomic profiles and pathway enrichment in LOAD subtypes

Following quality control and processing, the RNA-sequencing dataset comprised 483 cortical samples from 3 independent postmortem brain cohorts: UKBBN, PITT-ADRC, and ROSMAP. The dataset included 159 samples classified as LOAD-S1, with 45 from UKBBN, 61 from PITT-ADRC, and 53 from ROSMAP. LOAD-S2 consisted of 94 samples, with 31 from UKBBN, 21 from PITT-ADRC, and 42 from ROSMAP. The control group contained 193 samples, including 97 from UKBBN, 27 from PITT-ADRC, and 69 from ROSMAP.

**Fig. 4:**
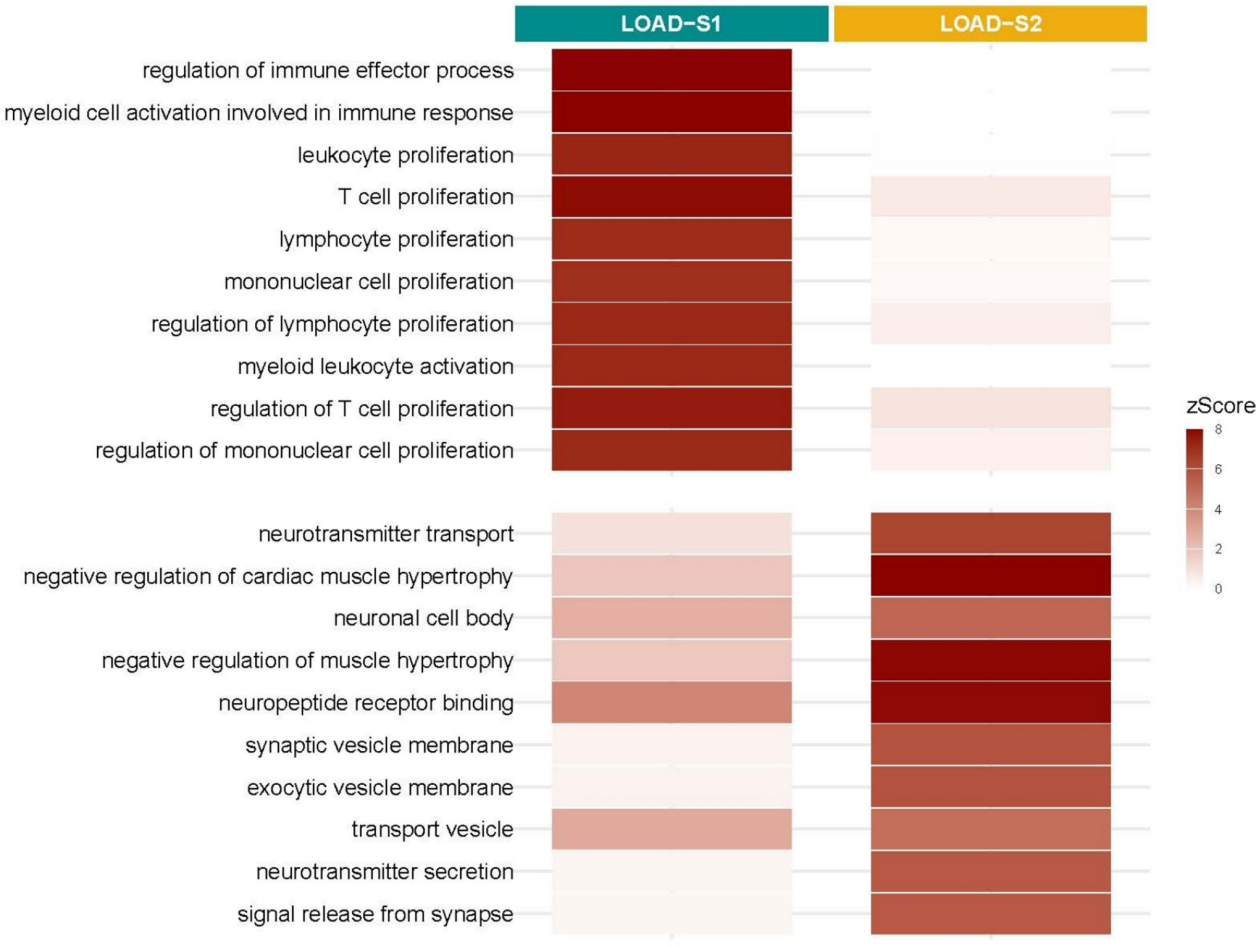
Transcriptomic characterization of LOAD epigenomic subtypes. Heatmap showing Z scores for the top 10 enriched Gene Ontology (GO) terms in LOAD-S1 and LOAD-S2 subtypes. *Z* scores indicate the deviation of observed gene counts from expected values, normalized by standard deviation. LOAD-S1 is enriched in immune-related pathways, reflecting a dominant immune/inflammatory signature, while LOAD-S2 shows enrichment in synaptic and neuronal pathways. Immune pathways prevalent in LOAD-S1 are demonstrating weak enrichment in LOAD-S2, and vice versa

Analysis of bulk PFC RNA-sequencing data identified 100 genes in LOAD-S1 and 120 in LOAD-S2 (nominal *p* value < 0.05 in all three cohorts, FDR-adjusted *p* value and FC > 1.5 in at least one, with a consistent direction of effect). (Supplementary Tables 16–17). GO enrichment analysis revealed that LOAD-S1 is driven by immune dysregulation and inflammation, with enriched terms including regulation of immune effector processes (GO:0002697), regulation of leukocyte proliferation (GO:0070663), and regulation of mononuclear cell proliferation (GO:0032944). In contrast, LOAD-S2 was enriched in synaptic and neuronal processes, including ‘synaptic vesicle membrane’ (GO:0030672), neurotransmitter transport (GO:0006836) and neuronal cell body (GO:0043025) (Supplementary Tables 18–19, Fig. 4).

### CpG–mRNA regulatory relationships across LOAD subtypes

Across cohorts, the majority of CpG–mRNA correlations were weak and inconsistent. However, a small subset of CpG–gene pairs met our robustness criterion, demonstrating reproducible methylation–expression associations across at least two cohorts. Notably, all replicated CpG–mRNA relationships were observed predominantly in the LOAD-S2 subtype, whereas no reproducible associations were detected for LOAD-S1. The replicated CpG–gene pairs in LOAD-S2 mapped to genes involved in biological processes central to AD pathology, including synaptic signaling and plasticity (YWHAE [19], CPEB1 [14], ITSN1 [17], PDE7B [29]), autophagy-lysosomal and proteostatic mechanisms (ATG16L2 [3]), and mitochondrial and metabolic function (CEROX1). In addition, several genes implicated in neuroimmune and glial-associated pathways (HLA-DPA1, HLA-DPB1, ARHGAP45, CMTM5, GPR37L1) were identified. The complete results are provided in Supplementary Table 20 and are visualized in Supplementary Fig. 9.

### Subtype-specific microglial single-nucleus transcriptomes and immune profiles

Analysis of snRNA sequencing data from the ROSMAP study explored the distinct microglial signatures associated with LOAD subtypes at the methylation level. This analysis focused on 12 microglial (MG) states, as characterized by Sun et al. [43] and was conducted by aggregating single-cell data into pseudobulk profiles for each MG state and subtype. After excluding states with insufficient cell numbers (< 100 cells in any group), only seven states (MG0–MG6) were retained for analysis (Supplementary Fig. 10). A one-way ANOVA followed by Tukey’s HSD post hoc tests (adjusted *p* < 0.05) identified MG4 state with a subtle but significant increase in proportional abundance in LOAD-S1 compared to controls, while the remaining states showed no statistically significant subtype-specific shifts (Supplementary Table 21).

DEA was restricted to the seven MG states (MG0–MG6) that met our minimum cell-count and read-depth criteria in both subtype and control samples. Across these states, sample sizes ranged from 13 to 46 for controls, 7–25 for LOAD-S1, and 6–20 for LOAD-S2. This analysis identified 269 unique genes meeting our lenient significance threshold (*p* < 0.05) in at least one subtype-to-subtype comparison and in a comparison versus healthy controls (Supplementary Table 22).

**Fig. 5:**
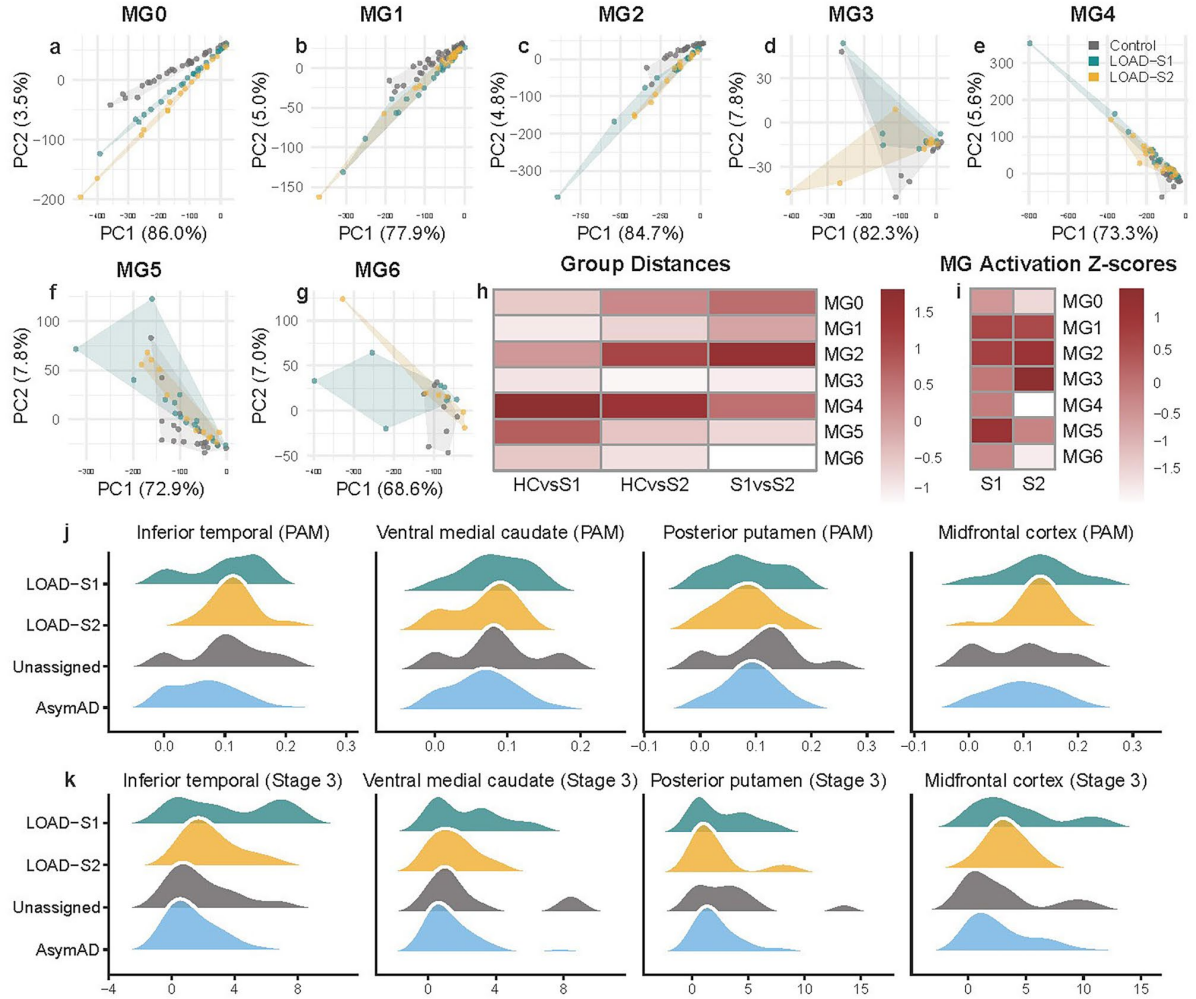
Subtype-specific microglial transcriptional and morphological activation states. PCA of pseudobulk microglial gene expression from single-nucleus RNA-seq data (ROSMAP) across seven microglial states (MG0–MG6), with LOAD-S1 and LOAD-S2 projected onto control-defined PCA spaces (**a**–**g**). The microglial states represent distinct functional programs, including homeostatic (MG0), neuronal surveillance (MG1), inflammatory I (MG2), ribosome biogenesis (MG3), lipid processing (MG4), phagocytic (MG5), stress signature (MG6) microglia. Pairwise Euclidean distances between healthy controls (HC), LOAD-S1 (S1), and LOAD-S2 (S2) summarize the magnitude of disease- and subtype-related transcriptional shifts within each microglial state (**h**). Panel **i** shows microglial activation *Z* scores, calculated from state-specific marker-gene expression and standardized relative to controls, highlighting subtype-dependent differences in activation across microglial states. Ridgeline (waterfall) density plots showing the distribution of **j** proportion of activated microglia (PAM) and **k** Stage 3 microglial pathology measures across four brain regions (inferior temporal cortex, ventral medial caudate, posterior putamen, and midfrontal cortex). Distributions are shown for LOAD-S1, LOAD-S2, Unassigned LOAD cases, and asymptomatic Alzheimer’s disease (AsymAD), highlighting differences in distributional shape and variability rather than mean shifts. Distributions are shown for LOAD-S1 (dark cyan), LOAD-S2 (dark goldenrod), Unassigned LOAD cases (grey), and (AsymAD; teal)

We next evaluated whether global transcriptional profiles within each microglial state differed between LOAD subtypes and controls by projecting samples into a PCA space derived from healthy microglia (Fig. 5a–g). Microglial state-specific modules (MG0-MG6) showed distinct patterns across healthy controls and the two LOAD subtypes. PCA of module eigengenes revealed that LOAD-S1 and LOAD-S2 samples shifted away from controls, most prominently in MG0, MG2, MG4.

PCA-based median distances indicated varying degrees of separation between microglial transcriptional profiles across diagnostic groups and microglial states. Across most clusters, distances between controls and LOAD subtypes were larger than those observed between LOAD-S1 and LOAD-S2, highlighting greater divergence of both LOAD subtypes from controls in PCA space (Supplementary Table 23; Fig. 5h). In several microglial states, LOAD-S2 showed greater separation from controls than LOAD-S1, indicating heterogeneity in the magnitude of subtype-associated microglial transcriptional shifts. Notably, in MG4, the separation between controls and LOAD-S2 exceeded that expected by chance (median distance = 76.43; 95% CI 5.14–64.06; empirical *p* = 0.0044; FDR = 0.031), indicating a pronounced divergence of LOAD-S2 from controls in this microglial state. Distances varied across microglial states, indicating that the extent of microglial divergence from controls is state dependent.

### Regional microglial morphology patterns across LOAD subtypes

Given the established relevance of microglial phenotype captured in LOAD subtypes, we next looked to test if quantitative morphology characterization of microglia in the ROSMAP cohort showed differences between subtypes. No pairwise comparisons survived FDR correction across regions or measures, indicating an absence of robust population-level differences in microglial activation between groups (all FDR-adjusted *p* > 0.05) (Supplementary Table 25). Effect sizes were predominantly small to moderate, particularly for comparisons between LOAD-S1 and LOAD-S2, reflecting substantial overlap in group distributions.

Despite the lack of statistical significance, consistent directional patterns were observed. For PAM measures, LOAD-S2 tended to show higher values than LOAD-S1 in the inferior temporal cortex, whereas LOAD-S1 more often exhibited higher PAM in subcortical regions, including the ventral medial caudate and posterior putamen, as well as in the midfrontal cortex. AsymAD generally showed lower PAM than symptomatic LOAD groups, particularly in cortical regions, although effect sizes were modest.

Directional effects were more pronounced for Stage 3 microglial pathology. Both LOAD-S1 and LOAD-S2 typically demonstrated higher Stage 3 values than AsymAD in cortical regions, most notably in the inferior temporal and midfrontal cortices, with moderate to large effect sizes in some comparisons. Subcortical regions were more heterogeneous, with Unassigned cases frequently showing higher Stage 3 pathology than both LOAD subtypes and AsymAD. Direct comparisons between LOAD-S1 and LOAD-S2 consistently yielded small effect sizes across regions, indicating minimal mean separation between methylation-defined subtypes.

Distributional inspection suggests greater variability in LOAD-S1, with broader PAM and Stage 3 distributions than LOAD-S2 across regions (Fig. 5j, k). In the inferior temporal cortex and ventral medial caudate, LOAD-S1 distributions extended further toward higher values, suggesting a subset of individuals with elevated microglial activation. In contrast, LOAD-S2 distributions were more compact and, in several subcortical regions, closely overlapped with AsymAD. Together, these findings indicate increased heterogeneity of microglial activation in LOAD-S1 rather than consistent differences in mean activation levels.

### Clinical and demographic characterization of LOAD subtypes

Across all cohorts, demographics, pathological and clinical assessments revealed no significant differences between LOADS1 and LOAD-S2 in terms of APOE status, polygenic risk scores for AD, age of onset, last cognitive assessment, or key measures of NFT and amyloid pathology (Supplementary Table 26). In the ROSMAP cohort, LOAD subtype status was not associated with differences in neuropathological measures, including Braak stage, neuritic and diffuse plaque burden, cerebral amyloid angiopathy, global AD pathology burden, TDP-43 pathology, cerebrovascular lesions, or Lewy body disease (Supplementary Table 27). These results suggest that the distinct epigenetic and transcriptomic signatures defining the subtypes, particularly their enrichment in specific cell-type DNAm patterns, operate independently of traditional clinical and pathological markers of AD.

## Discussion

This study identified two distinct epigenetic subtypes of LOAD by analyzing genome-wide DNAm profiles across three large-scale, independent postmortem brain cohorts. While these subtypes showed no association with established clinicopathological markers of LOAD, cellular and molecular characterization revealed distinct DNAm and transcriptional profiles, highlighting their biological relevance and potential role in LOAD heterogeneity.

Both subtypes exhibited enriched DNAm signatures in microglia only, indicating an immune and inflammatory association in both LOAD-S1 and LOAD-S2. However, bulk RNA sequencing analysis revealed that LOAD-S1 is primarily linked to immune and inflammatory pathways, whereas LOAD-S2 showed stronger associations with synaptic dysfunction and neuronal communication, highlighting distinct molecular mechanisms underlying these subtypes, which would be involving different functions within microglia. Consistent with the synaptic/neuronal GO enrichment observed for LOAD-S2, only reproducible CpG-mRNA associations were detected in LOAD-S2 and mapped to genes involved in synaptic signaling/plasticity, with additional links to proteostatic and metabolic pathways, suggesting a regulatory component to the LOAD-S2 molecular signature.

SnRNA-seq analysis of microglial states indicates that both LOAD-S1 and LOAD-S2 engage innate-immune biology within a shared AD background, but subtype differences are expressed primarily as state-dependent transcriptional shifts rather than large, uniform changes in microglial-state abundance. In line with this, marker-based “activation” differences between subtypes are generally modest, yet show consistent directional tendencies across states, suggesting that LOAD-S1 and LOAD-S2 may differentially weight partially overlapping microglial programs.

Within this framework, LOAD-S1 trends toward relatively greater engagement of inflammatory/innate-immune signaling in several microglial states, consistent with the broader immune-dominant bulk tissue signature and the greater inter-individual heterogeneity observed in microglial morphology. For example, elevated expression of innate-immune sensors and interferon-linked mediators such as UNC93B1 and STAT2 [15], together with reduced expression of regulatory “brakes” including PPAR-γ [12] and HSPA1B [27], is compatible with a shift toward more sustained inflammatory signaling. At the same time, modest induction of regulators such as PARP14 suggests that counter-regulatory mechanisms may also be engaged [16], underscoring that subtype differences likely reflect differences in balance rather than a binary switch.

By contrast, LOAD-S2 shows patterns consistent with relatively greater engagement of regulatory, clearance, and maintenance-associated programs in specific microglial contexts, alongside pronounced transcriptional remodeling in select states. Illustratively, increased PRKCE, VSIG4, and TGFB1 [4, 18, 24, 25], together with lower expression of pro-inflammatory coactivators such as EP300 and PTPN1 [49], aligns with microglial programs that may favor phagocytic function and tissue remodeling while tempering inflammatory amplification. Consistent with this interpretation, genes that map to specific microglial-state programs show subtype-dependent tendencies: for instance, we found that TYMP is more strongly downregulated in LOAD-S2 in MG2, whereas MGAT5 [40], which can support glycan-mediated regulation of immune receptor signaling, appears better preserved in LOAD-S2 than in LOAD-S1.

Overall, these results support a model in which LOAD-S1 is characterized by immune/inflammatory dominance and greater heterogeneity in microglial activation-related features, whereas LOAD-S2 is characterized by neuronal/synaptic dominance at the tissue level and, in microglia, more pronounced state-specific transcriptional remodeling despite only modest mean differences in activation scores between subtypes.

We observed co-localization between LOAD-S2-associated DMPs and AD risk variation at the SPI1 locus (Supplementary Table 15). SPI1 (PU.1) is a central myeloid transcription factor that shapes microglial identity and regulates broad microglial transcriptional programs, including immune-regulatory and phagocytic pathways [13]. In this context, SPI1-linked genetic regulation suggests a genomic link for LOAD-S2 and is consistent with our observation that LOAD-S2 shows pronounced, state-dependent microglial transcriptional remodeling with a relative weighting toward regulatory/clearance and maintenance-associated features.

The epigenomic-based LOAD subtypes identified in this study show notable correspondence with previously predicted molecular subtypes of AD. The bulk RNA‐seq characterization of epigenomics subtypes highlighted LOAD-S1 as immune/inflammatory and LOAD-S2 as synaptic/neuronal map remarkably onto the CSF proteomic subtypes of Tijms et al. [44], their “innate immune activation” subtype (Tijms S2) mirrors our LOAD-S1, and their “hyperplasticity” subtype (Tijms S1) recapitulates our LOAD-S2. In addition, we found that two out of the three categories identified in the transcriptomic-based subtypes of AD [33] assigned to immune-related and synaptic pathway share similar characteristics with the methylomic LOAD subtypes identified in this study. This cross‐platform concordance not only strengthens the biological validity of these subtypes, but also underscores that divergent microglial functions (pro‐inflammatory versus synapse‐supportive) are fundamental axes of heterogeneity in LOAD.

The presence of unassigned samples, which did not consistently align with LOAD-S1 or LOAD-S2 across cohorts, may represent mixed or intermediate phenotypes, exhibiting overlapping features of both LOAD-S1 and LOAD-S2 without strongly matching either subtype. Alternatively, these samples might reflect a cohort-specific subtype, driven by unique genetic or environmental factors that are less prevalent or absent in the replication cohorts. However, the Unassigned group is unlikely to represent random variation. It showed the greatest overlap with overall LOAD DMPs and with previously reported NFT-associated DMPs, suggesting a strong tangle-related methylation signal. Unassigned CpGs also lacked enrichment in annotated regulatory categories, consistent with increased heterogeneity less cell-type-specific localization of the DNAm changes. Although not statistically significant, regional morphology analyses showed trends toward higher Stage 3 microglial pathology in Unassigned cases than in either LOAD subtype across several subcortical regions. Future work should test whether Unassigned cases reflect mixed or intermediate phenotypes, differences in co-pathology burden, or cohort-specific influences, using larger datasets with harmonized neuropathology and cell-type-resolved multi-omic profiling.

The lack of correlation between LOAD subtypes and clinicopathological markers raises questions about how these epigenetic subtypes relate to traditional AD classifications or co-pathologies. However, prior research has demonstrated that neuroinflammation in AD is associated with disruptions in brain network connectivity, a key driver of disease progression that occurs independently of amyloid and NFT pathology or cortical atrophy [23]. Moreover, the lack of strong correlation with clinical or pathological markers in postmortem brain tissue has been consistently observed in transcriptomic-based AD subtypes conducted by Neff and colleagues [33]. This disconnect suggests that transcriptomic and epigenomic stratification may capture dimensions of disease biology that lie beyond the scope of current diagnostic criteria.

A key strength of this study is its large, cross-cohort design, analyzing postmortem brain samples from three independent biobanks (UKBBN, PITT-ADRC, and ROSMAP). This approach enhances the robustness of findings, ensuring validation across diverse populations while minimizing cohort-specific biases. In addition, the study integrates genetic, transcriptomic, and cell-type-specific data, providing a comprehensive characterization of the epigenomic-based LOAD subtypes. This multi-omic approach strengthens the biological relevance of the findings and offers insight into subtype-specific molecular mechanisms underlying AD heterogeneity.

A major limitation of this study is that while it provides robust evidence of epigenetic heterogeneity in LOAD and its association with distinct biological pathways, it does not establish causality. The relationship between epigenetic heterogeneity and LOAD is likely bidirectional, with DNAm changes both contributing to and resulting from the disease process. In addition, the lack of environmental risk factor data limits the ability to determine whether observed epigenetic changes stem from environmental exposures, lifestyle factors, or disease-related processes. The absence of detailed environmental data further restricts the ability to assess the role of non-genetic factors in driving the methylomic heterogeneity observed across LOAD subtypes.

## Conclusion

The findings of this study align with previous large-scale EWASs, which have shown that DNAm changes associated with AD predominantly occur in non-neuronal cells, particularly microglia [31, 41, 42, 52]. In this study, we expanded on these insights by identifying cell-type-specific DNAm signatures for the newly defined LOAD subtypes and establishing a clear link between these subtypes and distinct patterns of inflammatory microglial activity. We also showed that, although LOAD-S1 and LOAD-S2 samples are rigorously matched for age and overall AD pathology burden, they capture distinct microglial transcriptional states rather than successive disease stages. Even within brains carrying equivalent plaque and tangle loads, individual microglia can adopt divergent activation programs in response to local cues. These state-specific gene-expression patterns likely reflect microenvironmental heterogeneity, such as regional differences in amyloid conformation, local cytokine gradients, or neuron-glia cross-talk as well as cell-intrinsic epigenetic priming that persists despite similar pathological exposures. In other words, LOAD-S1 and LOAD-S2 define parallel neurodegeneration relevant processes, which, although representing a multi-cellular process, are each characterized in part by activation phenotypes of specific microglia co-existing in the AD brain. Data suggest these are not sequential snapshots of progression, given the inflammatory and synaptic gene programs emerging even when age and pathology are held constant.

This study further highlights the importance of subtype-specific analyses in uncovering heterogeneity within complex diseases like AD and provides a foundation for future research aimed at elucidating causal pathways and identifying potential therapeutic targets. While challenges remain regarding causality and environmental influences, these findings lay critical groundwork for refining precision medicine approaches in AD research and treatment.

## Supplementary Information

Below is the link to the electronic supplementary material.

Supplementary file1 (DOCX 3342 KB)

Supplementary file2 (XLSX 389 KB)

## Data Availability

The PITT-ADRC datasets used in this study are available on Synapse (https://www.synapse.org/) under Synapse ID: syn23538600. Access requires creating a Synapse user account and submitting a data access request. The UKBBN dataset is accessible via GEO under accession number GSE284764. ROSMAP datasets are also deposited on Synapse (Synapse IDs: syn7357283, syn23650893, syn3157325, syn25006903). Microglial snRNA sequencing data and markers of microglial states were obtained from https://compbio.mit.edu/microglia_states/. The ROSMAP mQTL dataset is accessible at https://mostafavilab.stat.ubc.ca/xqtl/. All codes used for DNA methylation and bulk transcriptomic analyses, clustering, replication, and cross-cohort validations are available at https://github.com/Dementia-Systems-Biology/LOAD_subtyping.
