# Supplementary material for: Epigenomic subtypes of late-onset Alzheimer’s disease reveal distinct microglial signatures
Source: Acta Neuropathol. 2026 Feb 24;151(1):20. doi: 10.1007/s00401-026-02990-y (PMC12932393; doi:10.1007/s00401-026-02990-y)
Supplement: Supplementary file 1 — Supplementary file1 (DOCX 3342 KB) [file 401_2026_2990_MOESM1_ESM.docx]

**K-means clustering**

**Hierarchical clustering**


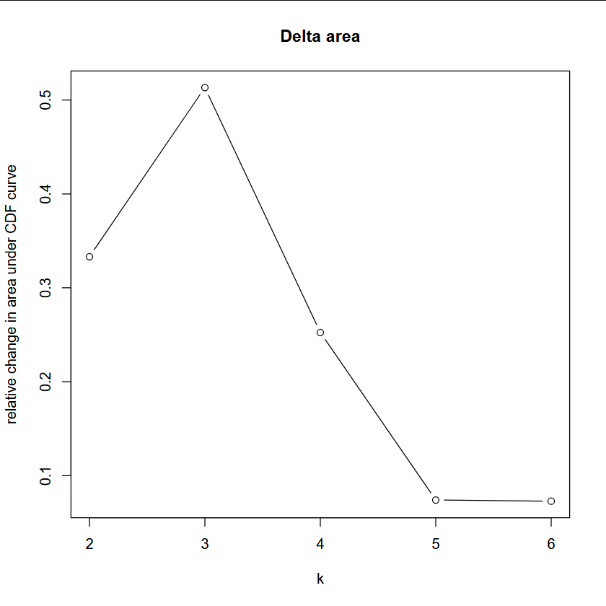

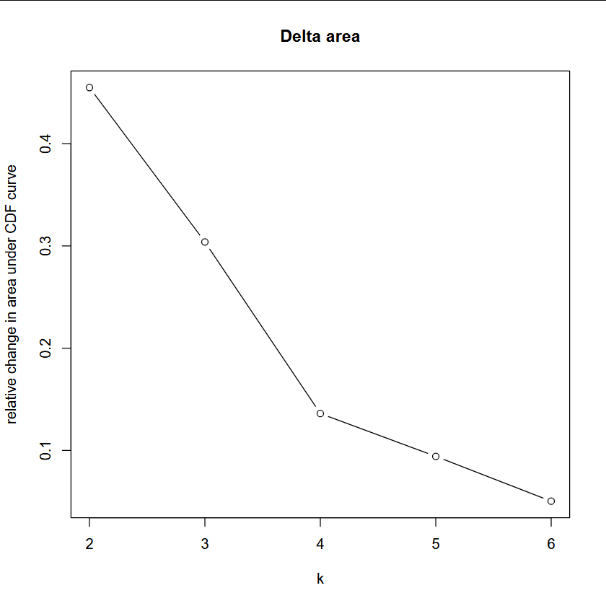


**ROSMAP**

**PITT-ADRC**

**UKBBN**


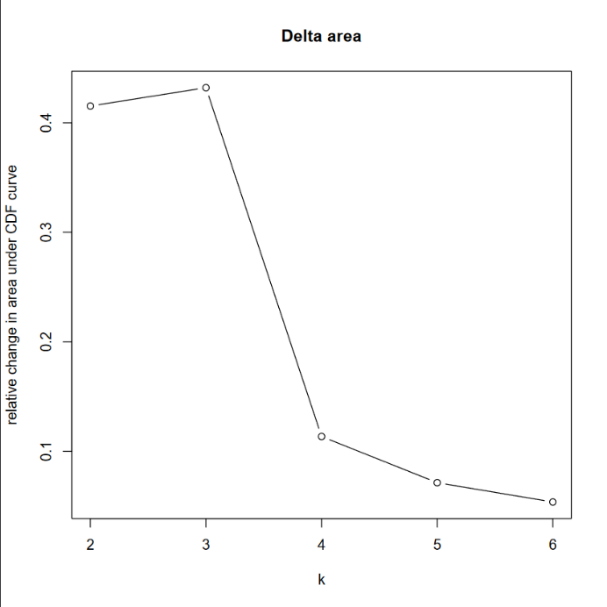

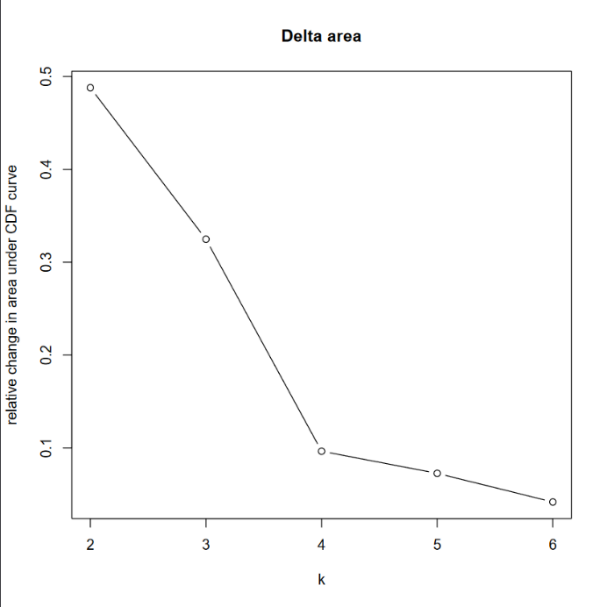

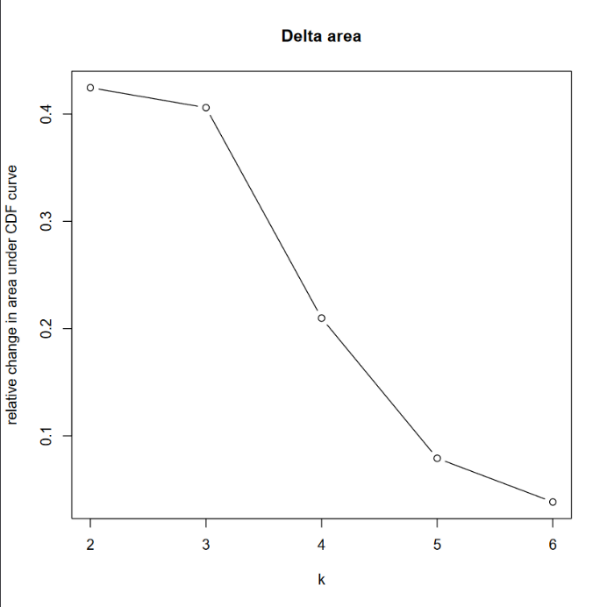

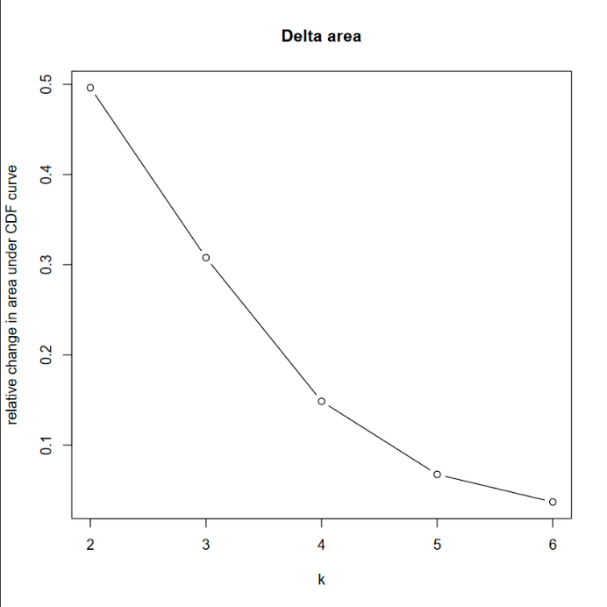


**Supplementary Figure 1. Optimal cluster determination using the Elbow method.** Plots illustrate the application of the Elbow method to select the optimal number of clusters using Hierarchical (Left) and K-means (Right) for each cohort.


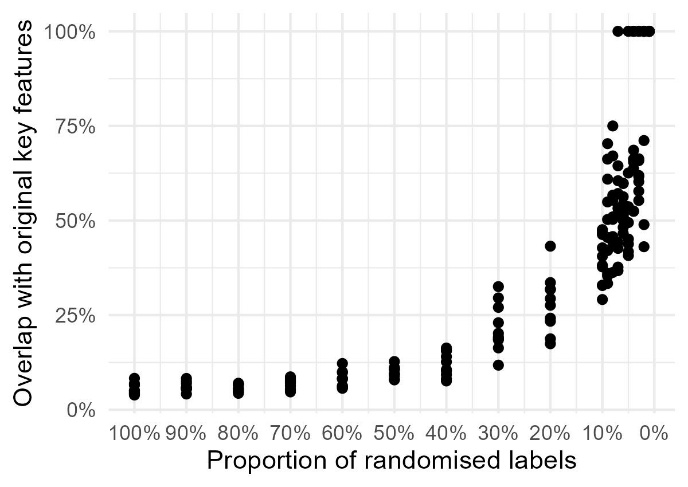

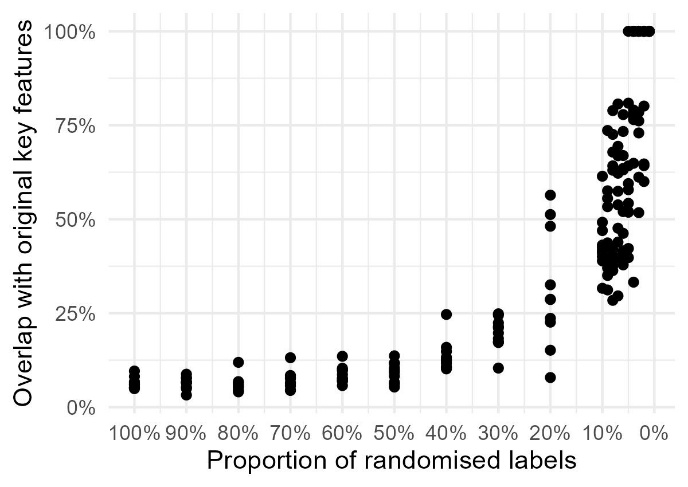

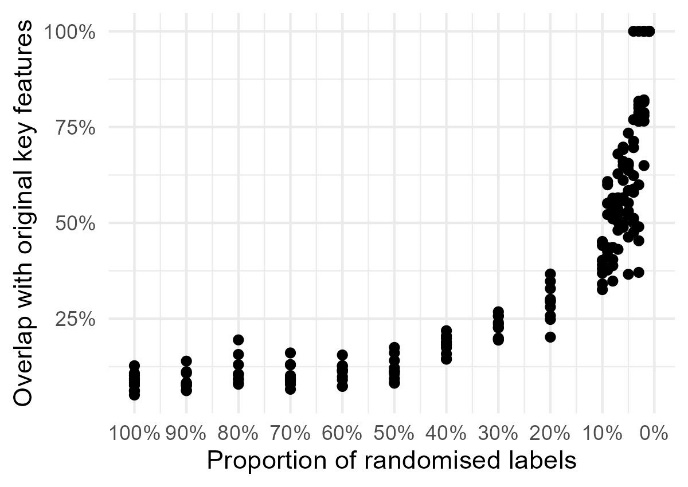

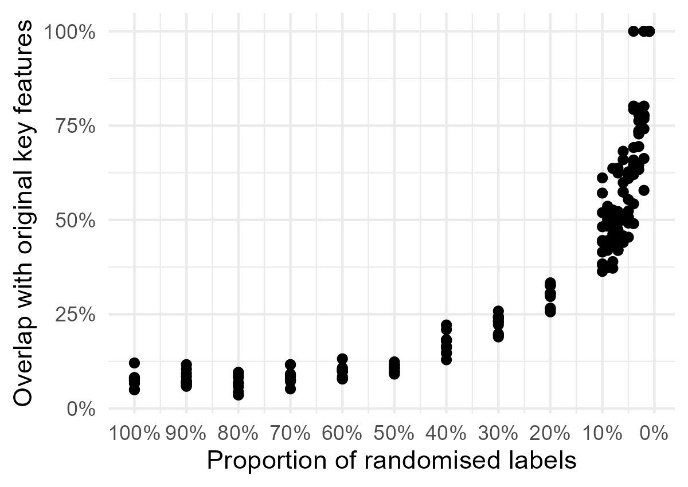

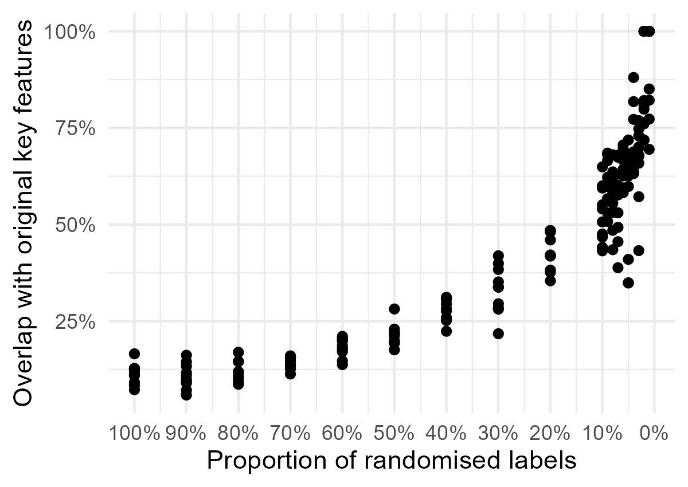

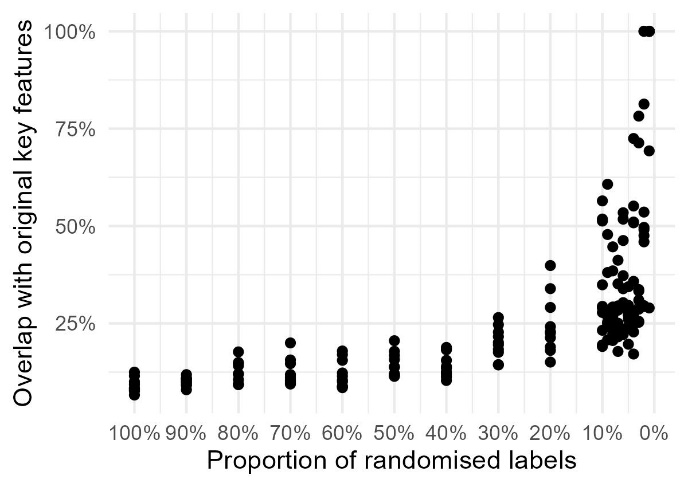


**Hierarchical clustering**

**K-means clustering**

**ROSMAP**

**UKBBN**

**PITT-ADRC**

**Supplementary Figure 2. Validation of cluster specificity through label randomization.** Clusters were validated by randomizing cluster labels across varying proportions of the sample populations. A minimal overlap between discriminative features of the original clusters and those identified using randomized labels were observed, particularly when randomization was applied to the full dataset.

**
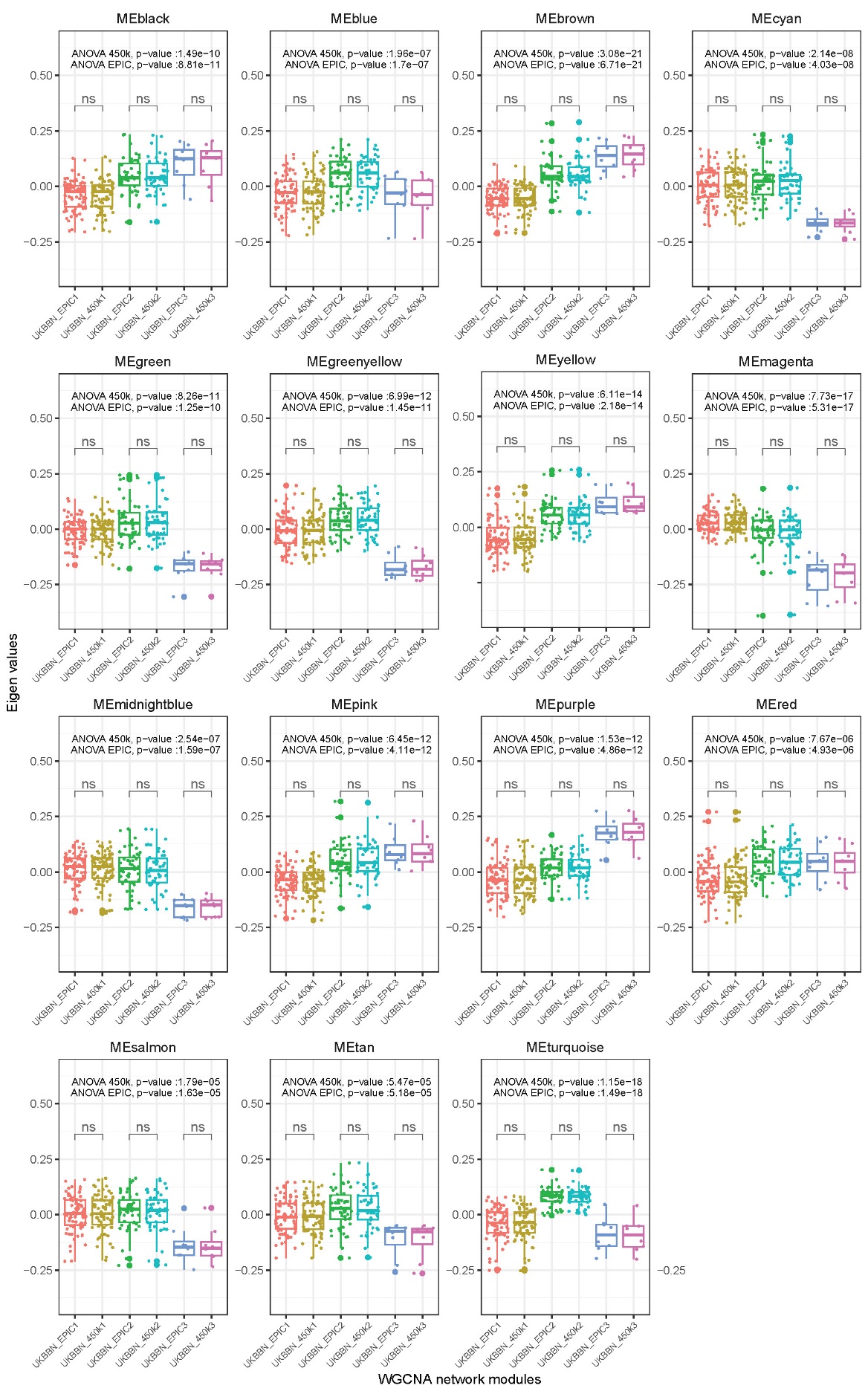
**

**Supplementary Figure 3. Assessment of DNAm array compatibility in the UKBBN using hierarchical clustering.** The significant relationships between module eigenvalues and the three identified clusters, as determined by ANOVA using EPIC arrays, and the subset of probes available on 450K arrays.


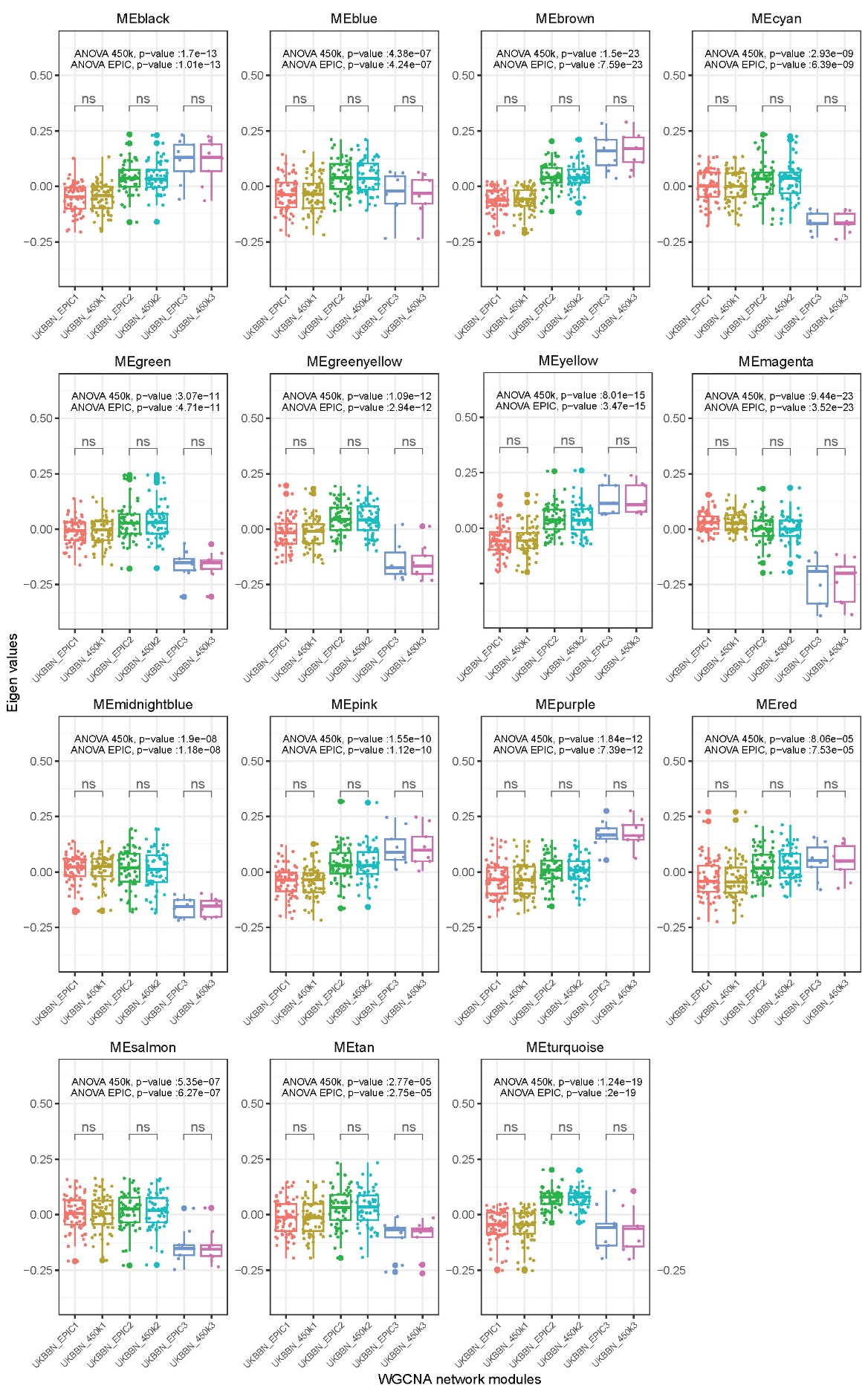


**Supplementary Figure 4. Assessment of DNAm array compatibility in the UKBBN using K-mean clustering.** The significant relationships between module eigenvalues and the three identified clusters, as determined by ANOVA using EPIC arrays, and the subset of probes available on 450K arrays.


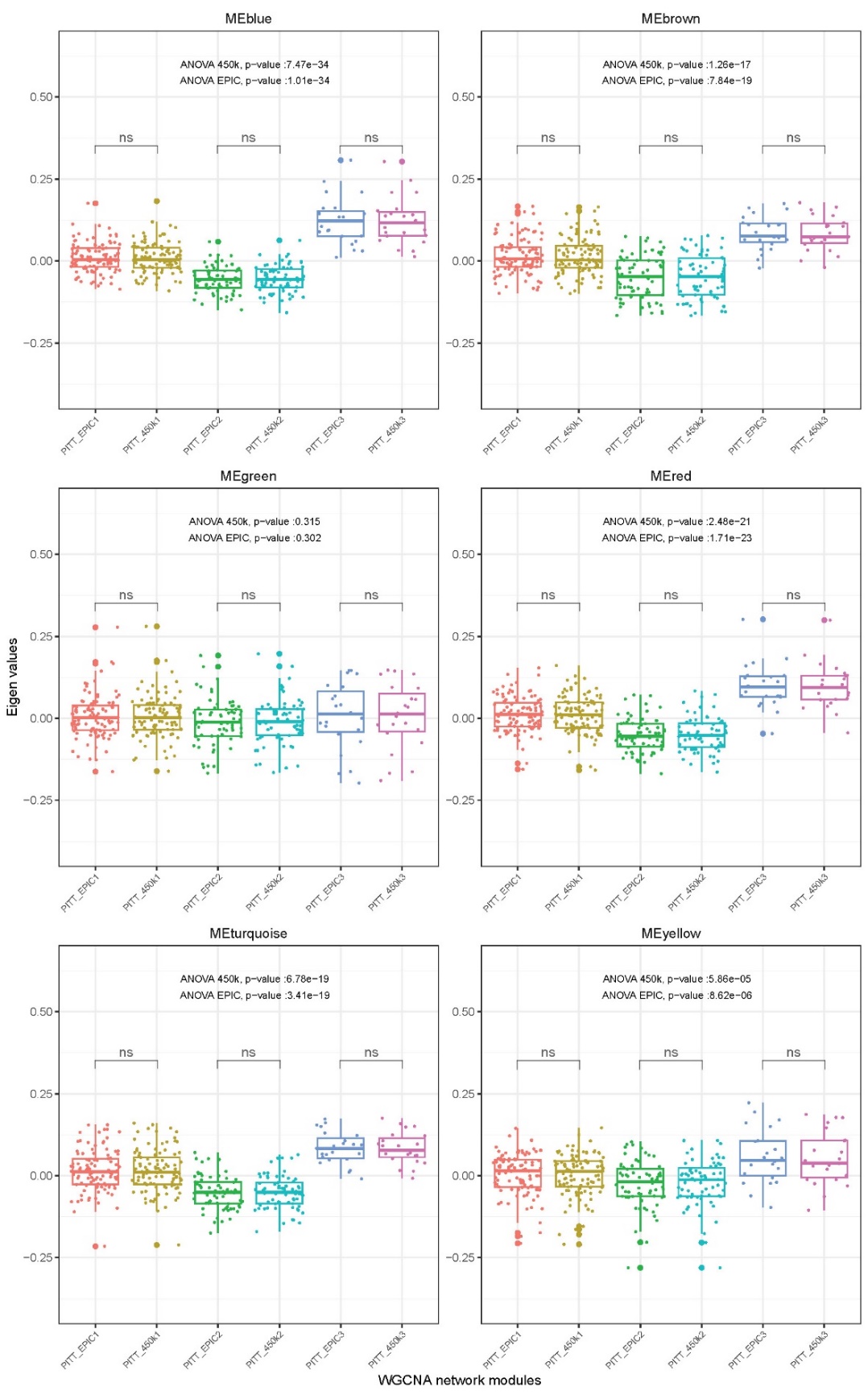


**Supplementary Figure 5. Assessment of DNAm array compatibility in the PITT-ADRC using hierarchical clustering.** The significant relationships between module eigenvalues and the three identified clusters, as determined by ANOVA using EPIC arrays, and the subset of probes available on 450K arrays.


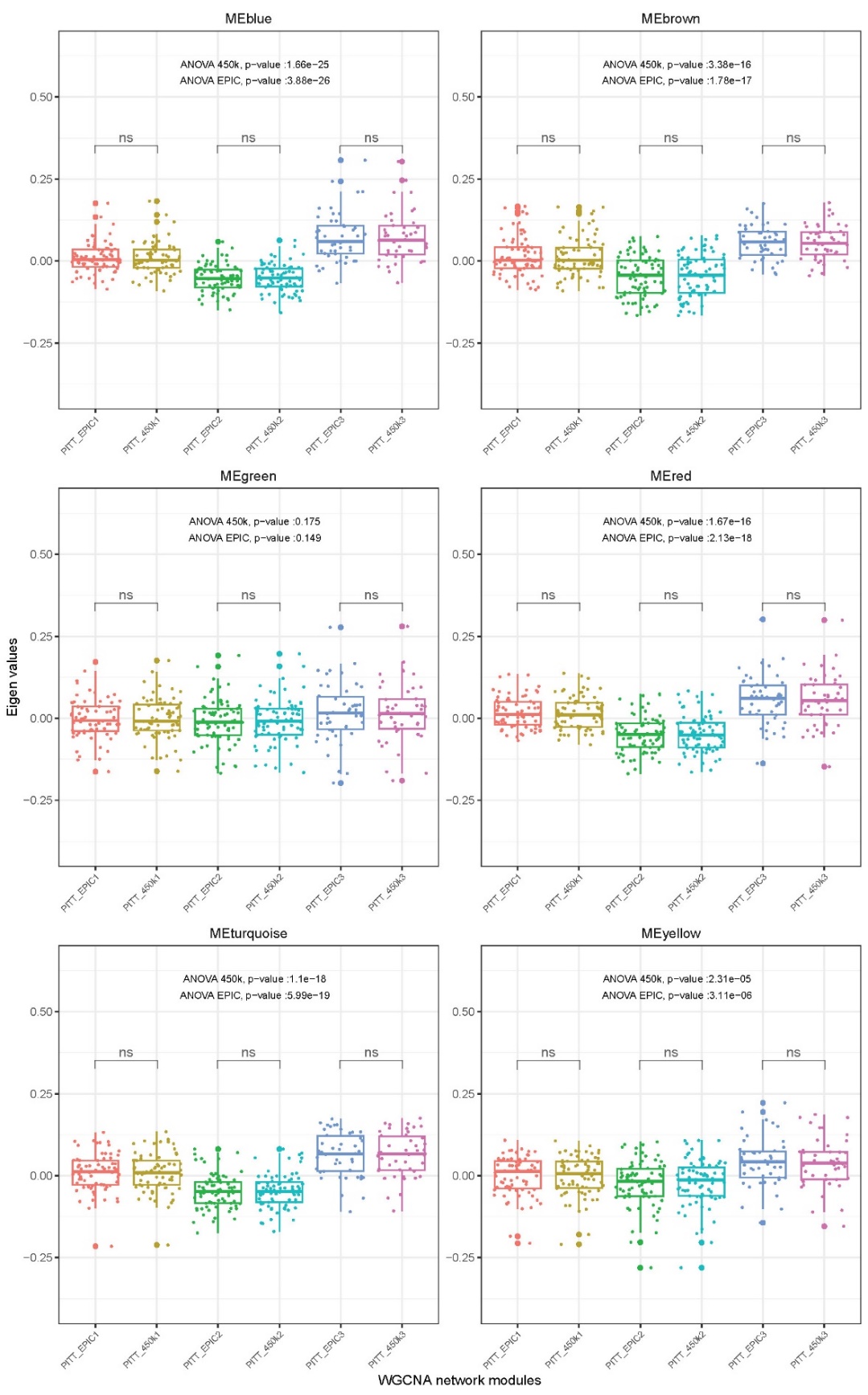


**Supplementary Figure 6. Assessment of DNAm array compatibility in the PITT-ADRC using K-means clustering.** The significant relationships between module eigenvalues and the three identified clusters, as determined by ANOVA using EPIC arrays, and the subset of probes available on 450K arrays.


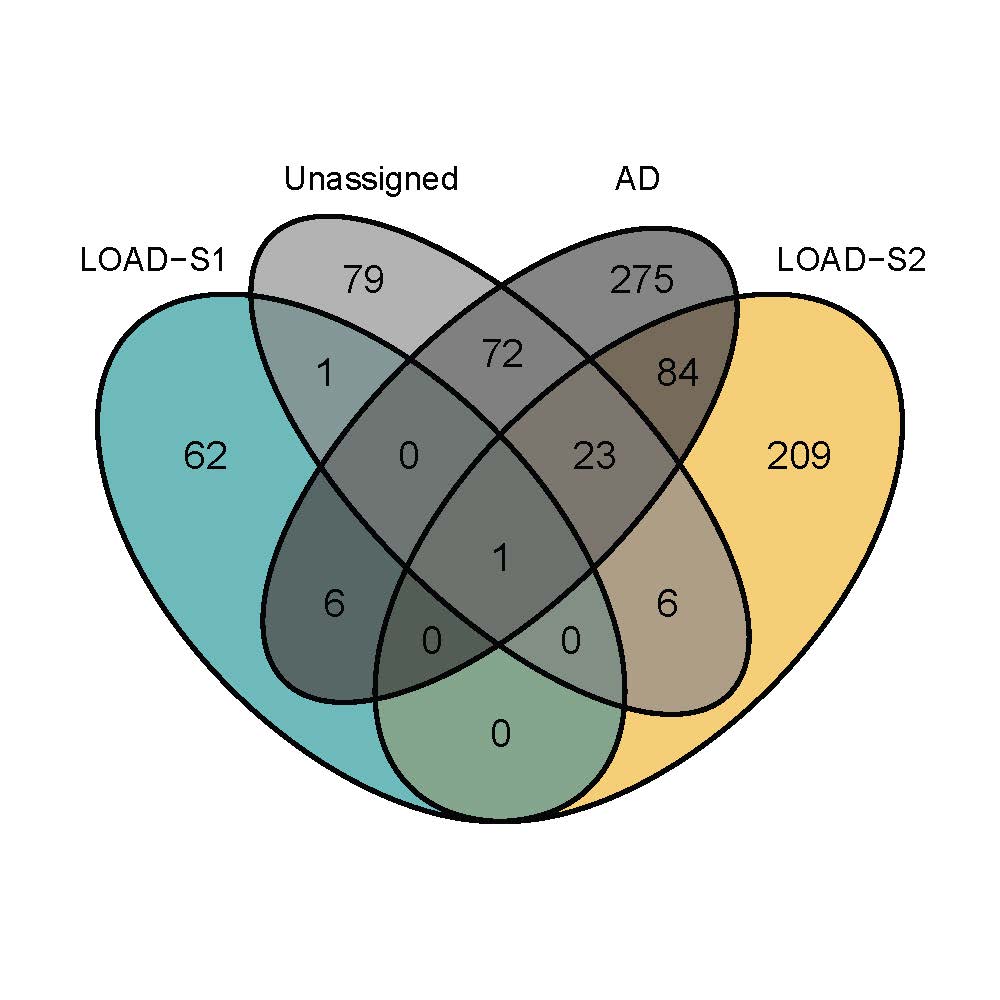


**Supplementary Figure 7. Distinct and Shared DMPs across LOAD, LOAD-S1, LOAD-S2, and Unassigned Groups.** Venn diagram illustrates the overlap of differentially methylated positions (DMPs) associated with LOAD-S1, LOAD-S2, and the Unassigned group relative to overall LOAD. Minimal overlap is observed between LOAD-S1 and LOAD-S2, highlighting their distinct methylomic profiles.


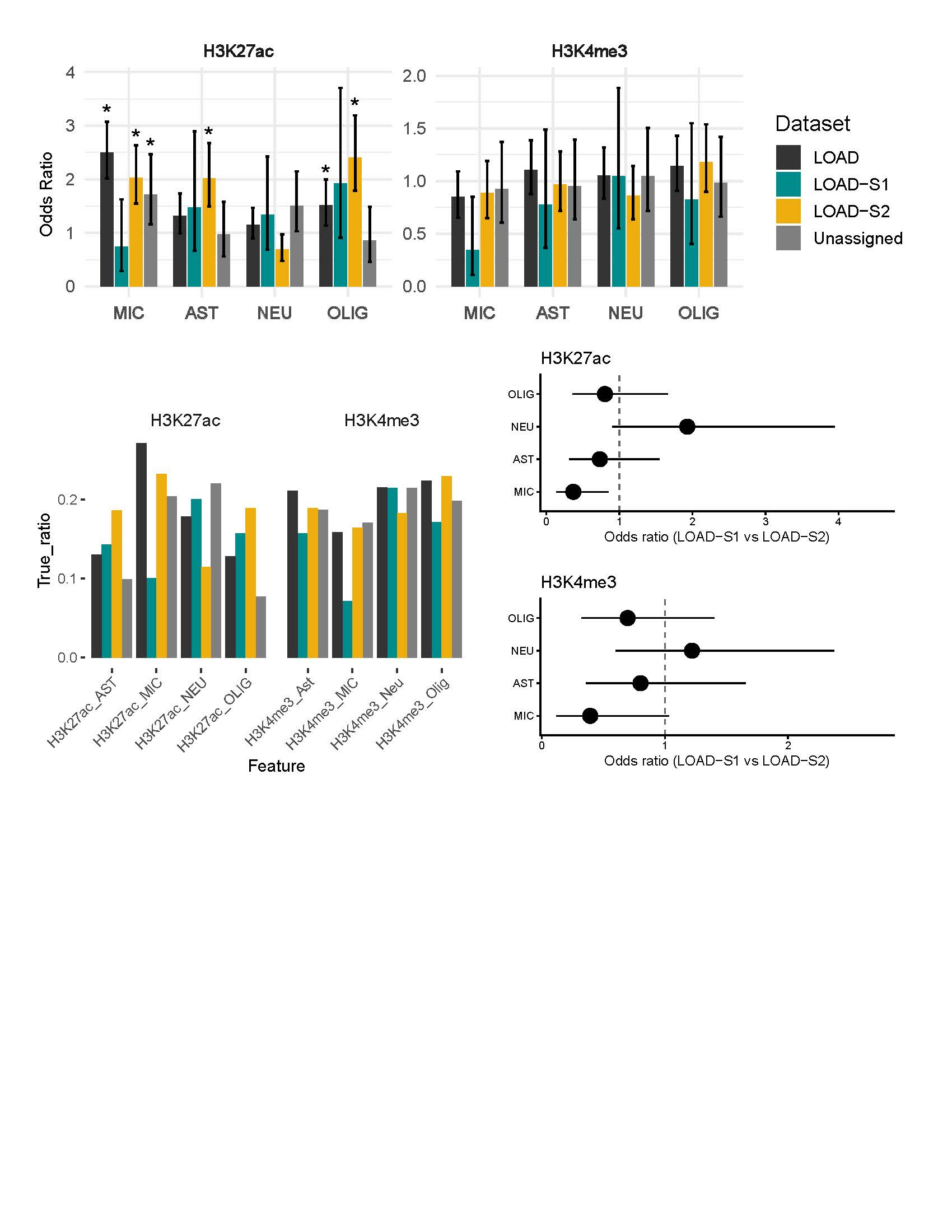


**Supplementary Figure 8. Cell type–specific regulatory annotation of subtype-specific CpGs.** Odds ratios with 95% confidence intervals from Fisher’s exact tests comparing LOAD-S1 and LOAD-S2 are shown for each cell type (upper panels). Statistical significance after false discovery rate (FDR) correction is indicated (*FDR < 0.05;). Bar plots (bottom-left panels) depict the proportion of loci annotated with H3K27ac, H3K4me3, across datasets. Forest plots display odds ratios (95% confidence intervals) from Fisher’s exact tests comparing LOAD-S1 and LOAD-S2. OR > 1 indicates enrichment in LOAD-S1, and OR < 1 indicates enrichment in LOAD-S2 (bottom-right panels).


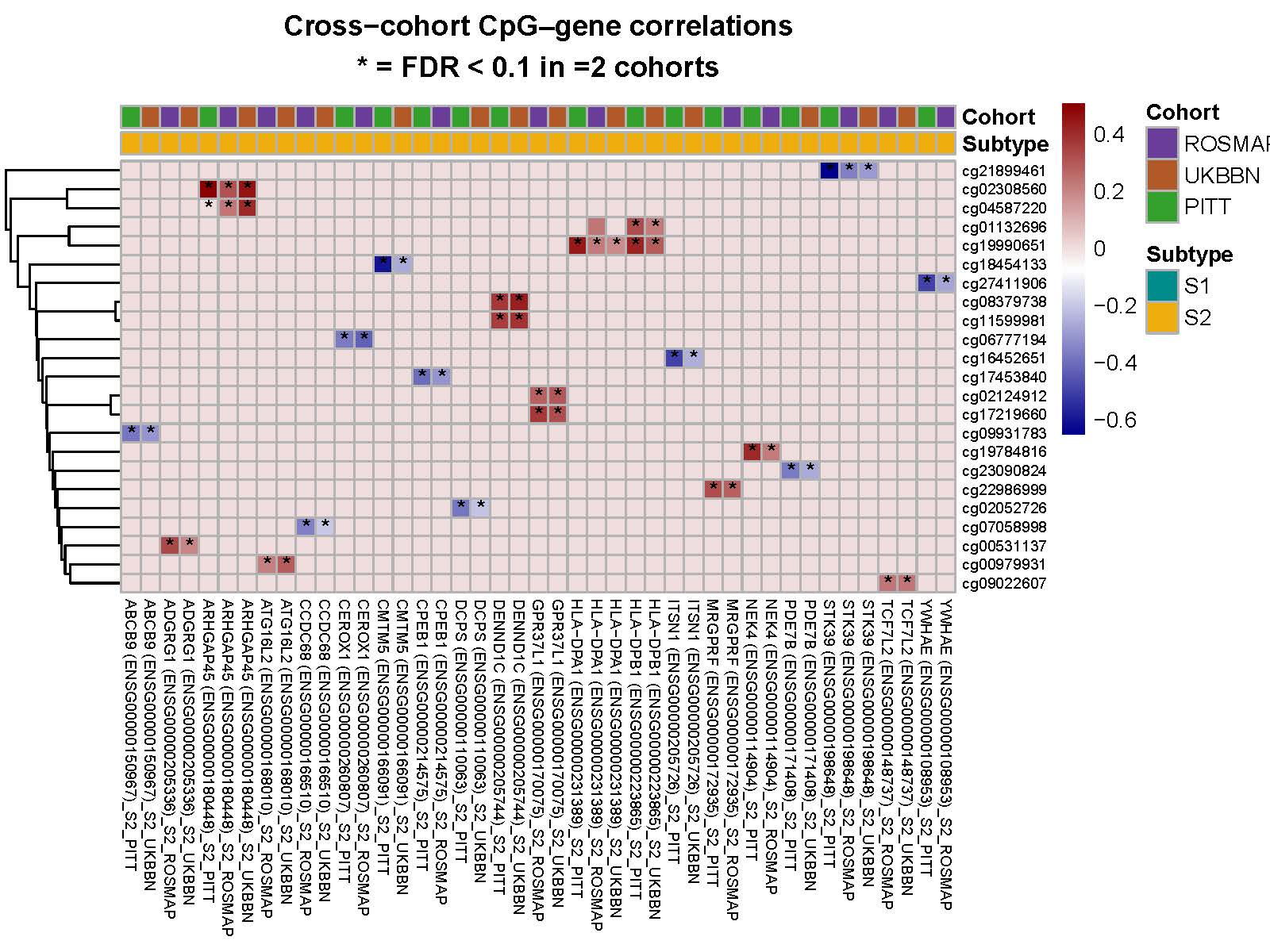


**Supplementary Figure 9. Cross-cohort CpG–gene expression correlations within LOAD subtypes.** Heatmap showing Spearman correlations between subtype-specific differentially methylated CpGs and the expression of their promoter-mapped genes (TSS ±10 kb), computed separately within LOAD-S1 and LOAD-S2 across the ROSMAP, UKBBN, and PITT-ADRC cohorts. CpG-gene pairs shown met the significance threshold of FDR < 0.1 in at least two cohorts (indicated by asterisks). Rows represent CpGs and columns represent gene–cohort combinations, hierarchically clustered based on correlation patterns. Color intensity reflects correlation strength and direction, providing direct evidence of coordinated subtype-specific epigenetic and transcriptomic regulation.


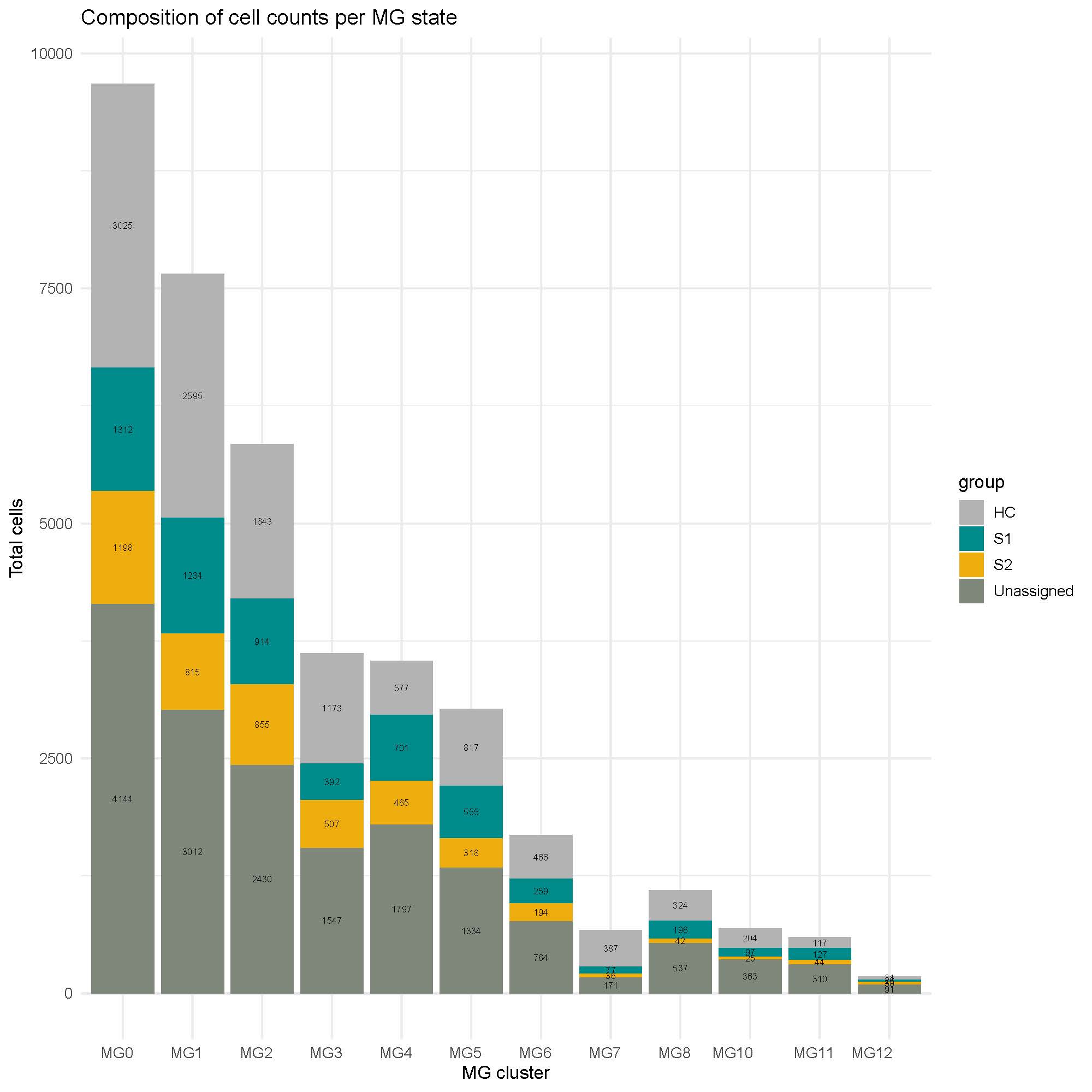


**Supplementary Figure 10. Distribution of microglial cell counts across MG states and diagnostic groups.** Total numbers of microglial cells assigned to each MG state (MG0–MG12) are shown for control (HC), LOAD-S1, LOAD-S2, and unassigned groups. Cell counts are reported descriptively and reflect the aggregated number of cells per MG state and group.
